# Trade-driven relocation of air pollution and health impacts in China

**DOI:** 10.1038/s41467-017-00918-5

**Published:** 2017-09-29

**Authors:** Haikun Wang, Yanxu Zhang, Hongyan Zhao, Xi Lu, Yanxia Zhang, Weimo Zhu, Chris P. Nielsen, Xin Li, Qiang Zhang, Jun Bi, Michael B. McElroy

**Affiliations:** 1 State Key Laboratory of Pollution Control and Resource Reuse, School of the Environment, Nanjing University, Nanjing, 210023 China; 2 John A. Paulson School of Engineering and Applied Sciences, Harvard University, Cambridge, MA 02138 USA; 3 Department of Earth System Science, Tsinghua University, Beijing, 100084 China; 4 School of Environment and State Key Joint Laboratory of Environment Simulation and Pollution Control, Tsinghua University, Beijing, 10084 China; 5 State Environmental Protection Key Laboratory of Sources and Control of Air Pollution Complex, Beijing, 100084 China; 6 Department of Earth and Planetary Sciences, Harvard University, Cambridge, MA 02138 USA

## Abstract

Recent studies show that international trade affects global distributions of air pollution and public health. Domestic interprovincial trade has similar effects within countries, but has not been comprehensively investigated previously. Here we link four models to evaluate the effects of both international exports and interprovincial trade on PM_2.5_ pollution and public health across China. We show that 50–60% of China’s air pollutant emissions in 2007 were associated with goods and services consumed outside of the provinces where they were produced. Of an estimated 1.10 million premature deaths caused by PM_2.5_ pollution throughout China, nearly 19% (208,500 deaths) are attributable to international exports. In contrast, interprovincial trade leads to improved air quality in developed coastal provinces with a net effect of 78,500 avoided deaths nationwide. However, both international export and interprovincial trade exacerbate the health burdens of air pollution in China’s less developed interior provinces. Our results reveal trade to be a critical but largely overlooked consideration in effective regional air quality planning for China.

## Abstract (web summary)

International and domestic interprovincial trade of China are entangled, but their health impacts have been treated separately in earlier studies. Here Wang. quantify the complex impacts of trade on public health across China within an integrative framework.

## Introduction

The Chinese economy has experienced rapid growth since the open door reform policy was adopted in 1978. A significant portion of the growth has been driven by exports, particularly after China became a member of the World Trade Organization in 2001. In 2010, China became the world’s second largest economy and the gross domestic product (GDP) reached US$5.88 trillion, of which 27% was contributed by merchandise exports^1^. As the world’s largest exporter, China accounted for over 10% of world merchandise export value in 2009^2^. Although global trade in principle benefits both trading nations in terms of GDP, it tends to outsource pollution to less developed nations by taking advantage of low-cost labor and energy as well as lax environmental enforcement to increase returns on capital^3^. Because China’s economic boom has been fueled primarily by coal using less advanced technologies for production and emission control^4^, international exports have become an increasingly important factor driving China’s air pollutant emissions in recent decades^5^.

China is a vast country with substantial disparities across provinces in terms of resource and energy endowments, economic development, population densities, and lifestyles, resulting in trade between provinces of goods with embodied pollution. In recent years, emissions grew rapidly in some interior provinces but stabilized or even decreased in many coastal regions^6^, partly because interior provinces export emission-intensive products (e.g., raw materials or energy) to support production and consumption of finished goods in coastal regions^7, 8^. At the same time, emissions in interior provinces can contribute to air pollution in coastal provinces through transport of both primary and secondary pollutants, the latter formed by chemical reactions of precursor gases in the air^9^. Provinces often cite this quandary in disputes about respective responsibilities in regional air quality planning in China. Note that the health effects of air pollution exposures are dominated by those due to fine particles (PM_2.5_), which take many chemical forms and occur both as primary pollutants and secondary products of SO_2_, NO_X_, NH_3_, and other gases.

A number of studies have applied input−output models to analyze CO_2_ emissions embodied in international and Chinese interprovincial trade^7, 10, 11^. Application of these methods to study trade-related air pollution in China is an emerging field with important implications. Zhao et al.^8^ estimated that 15–23% of China’s air pollutant emissions were related to exports for foreign consumption. Lin et al.^12^ investigated air pollution embodied in China’s international trade and found its exports responsible for 23–34% of the concentrations of sulfate, 10–23% of black carbon, and 12–23% of CO over East China in 2006. Jiang et al.^13^ estimated that international exports caused 12% of the total mortality attributable to PM_2.5_ across China in 2007. Zhang et al.^14^ proposed a comprehensive framework to differentiate the total mortality of ambient PM_2.5_ according to the source emissions generated in local production, international exports, and international imports for various regions in the world. They indicated that consumption-based premature deaths due to PM_2.5_ in China (835,110) are much lower than production-based ones (1,023,689). However, existing studies of trade-related air pollution in Chinese provinces are not linked with a global multi-regional input−output (MRIO) model, thus are less accurate in quantifying the impact of international trade, both generally and with regard to specific countries, on China’s air pollution. Study of the impact of interprovincial trade on air pollution, moreover, is critical for designing regional air quality improvement plans in China, but has not yet been addressed in the literature. For a thorough quantitative understanding of the effects of trade on regional air pollution and public health across China, it is essential to integrate interprovincial and international trade into one MRIO model.

In this study, we calculate the extent to which international and interprovincial trade resulted in relocation of air pollutant emissions across 30 Chinese provinces (not including Tibet, Hong Kong, Macao, and Taiwan) and 40 other countries/regions in 2007, and we evaluate the subsequent effects on air quality and public health across China. We accomplish this by linking a global integrated MRIO model, an air pollutant emissions inventory, an atmospheric chemical transport model, and an exposure-response model. We show that nearly 208,500 premature deaths resulting from PM_2.5_ pollution throughout China are attributable to international exports. Interprovincial trade has mixed environmental impacts across China. It led to improved air quality in developed coastal provinces and avoided an estimated 78,500 deaths nationwide in 2007. However, both international trade and interprovincial trade exacerbate the health burdens of air pollution in China’s less developed interior provinces, where large quantities of emission-intensive goods are manufactured for direct consumption in the coastal provinces or exported indirectly in the form of raw materials used in the supply chain of exported finished goods.

## Results

### Impact of international exports

**Fig. 1:**
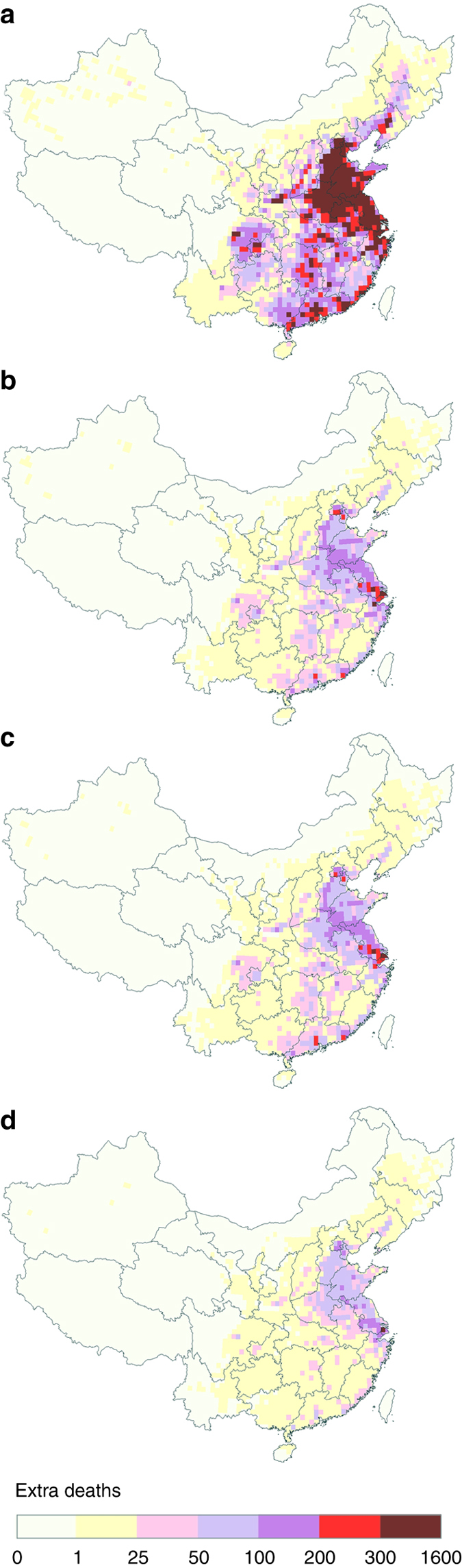
Premature deaths attributable to ambient PM_2.5_ associated with air pollutant emissions embodied in international exports from China. Mortalities attributable to: **a** total international exports; **b** exports to Europe; **c** exports to North America; and **d** exports to East Asia

As illustrated in Fig. 1, emissions resulting from production of goods exported internationally from China contributed to degraded ambient air quality (Supplementary Fig. 1b), increasing health burdens over nearly all of China. It increased the estimated national population-weighted mean PM_2.5_ concentration (PWM-PM_2.5_) by nearly 20%, from 45.5 to 57.6 μg/m^3^ in 2007. The estimated mortality associated with pollution embodied in international exports was 208,500 throughout China, with a 95% confidence interval (CI95) of 158,800–255,900 (Fig. 1a), 150% higher than the recorded Chinese traffic fatalities in the same year. The European Union, North America, and East Asia, the largest three importing regions for Chinese goods (at 24, 21, and 13% of total export value, respectively), were associated with an estimated 47,000, 45,100 and 27,100 premature deaths in China from PM_2.5_ exposures in 2007, respectively (Fig. 1b–d).

**Fig. 2:**
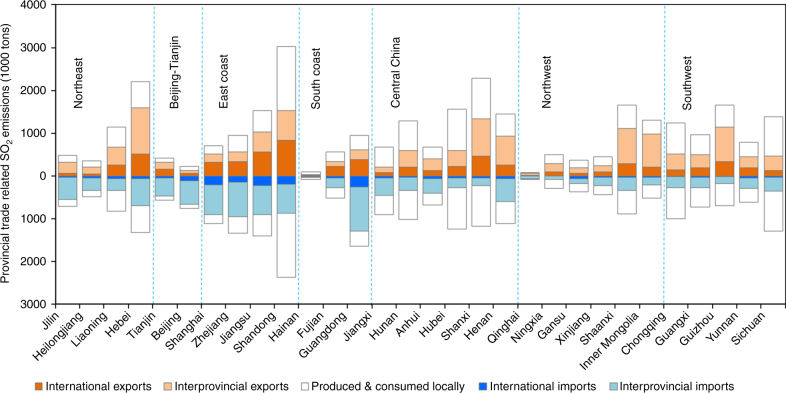
Comparison of provincial air pollutant emissions from production and consumption perspectives. The production-based and consumption-based emissions of SO_2_ by province in 2007 are illustrated on top and bottom, respectively. Note that the two white fields for each province are identical as they represent the same quantity

Very high emission intensity (i.e., emissions per unit of economic output) drives the health impacts attributable to pollution embodied in China’s international exports. While in value terms China’s imports (US$955 billion) in 2007 were ~ 80% of its exports (US$1.22 trillion) and had similar sector composition (Supplementary Fig. 2), emissions derived from international imports were far less than those from international exports (e.g., 30% for emissions of SO_2_, a key precursor gas of secondary PM_2.5_) (Fig. 2 and Supplementary Fig. 3). This imbalance highlights the difference in emission intensities of exported and imported goods (Supplementary Table 1; note that missing data for a few sectors of countries other than China might increase the magnitude of the above-mentioned difference in air pollutant emissions). For example, the SO_2_ emission intensity of China’s exports was as high as 5.4 g SO_2_ US$^−1^ in 2007, 2.3 times that of imports. As a result, international export-related SO_2_ emissions reached 6.97 Mt, accounting for 23% of China’s total production-based emissions. Again, these emissions were mainly associated with exports to the European Union (23%), North America (21%), and East Asia (12%) (Supplementary Fig. 4).

Our results further demonstrate that the impacts of international exports of Chinese goods on air pollution and public health are unevenly distributed in China (Fig. 1a and Supplementary Fig. 1b). Thanks to an advantageous export structure and deployment of more advanced production and air pollution control technologies^15^, the coastal provinces, including Shanghai and Guangdong, were responsible for 40% of export values in 2007 but only 11% of China’s total mortality associated with emissions embodied in international exports (~ 22,500 deaths). In contrast, exports from China’s interior regions include primarily energy-intensive and emission-intensive products with lesser monetary values. Furthermore, the production of export goods often requires inputs of raw materials and energy forms produced by heavy industry (i.e., metals, mining, coke and refined petroleum, chemicals, non-metal mineral products, and coal-fired electricity), which are geographically concentrated in China’s interior provinces^7^. These sources increased indirect export-related emissions (i.e., those arising from production of goods and services that are not directly exported from the country but used in the production of final export goods) from the interior provinces. Despite contributing only 19.0% of China’s total export value, the interior provinces suffered much higher air pollution burdens from exports, e.g., 57.9% of related SO_2_ emissions. For interior regions with large populations (e.g., central China, home to 360 million people), the health burden associated with international exports can be quite high in some provinces including Henan (18,000 deaths), Hebei (15,600), and Anhui (13,300).

While the above emphasizes trade-derived air pollution and related health impacts in China, it is important to acknowledge that other countries suffer health impacts of air pollution from emissions embodied in exports to China. As reflected in the disparities of embodied emission intensities of exports vs. imports, however, the total environmental impacts resulting from imports to China will be considerably less than those resulting from its exports. It should also be noted that because international exports have at least partially replaced domestic consumption, our counterfactual scenarios excluding emissions embodied in exports might overestimate the health impact on China of its international exports. On the other hand, production for international export may also prompt diffusion of improved technologies to or within China, in which case its effect on the health impact within China might be underestimated.

### Impact of interprovincial trade

Compared to air pollutant emissions from international trade, those embodied in trade between China’s provinces were even greater in 2007 (Fig. 2 and Supplementary Fig. 3). These emissions were mainly driven by the concentration of consumption in coastal regions while production is concentrated in interior provinces through domestic supply chains (Supplementary Fig. 5). For example, over 60% of the consumption-based SO_2_ emissions in Beijing−Tianjin, the Yangtze River Delta, and the Pearl River Delta were generated outside of their boundaries, in other provinces. Interior provinces, where per capita GDP averages less than $3000 compared to $4400−$8700 in coastal regions, exhibit the largest difference in the emission intensity of exports and imports, resulting in nearly 75% of the interprovincial trade-related emissions. For example, in Guizhou, where the per capita GDP was only $969 in 2007, the SO_2_ emission intensity of interprovincial exports was 6.6 times that of imports from other provinces. Ratios over 2 exist in other less developed provinces, including Shanxi, Ningxia, and Inner Mongolia. Even for production of the same products, emission intensities of the interior provinces are usually higher than their coastal counterparts because of different energy structures and technology levels. The coal-fired electric power industry generates 58.4 g SO_2_ per US$ of electricity in Inner Mongolia, nearly twice that in Shanghai.

**Fig. 3:**
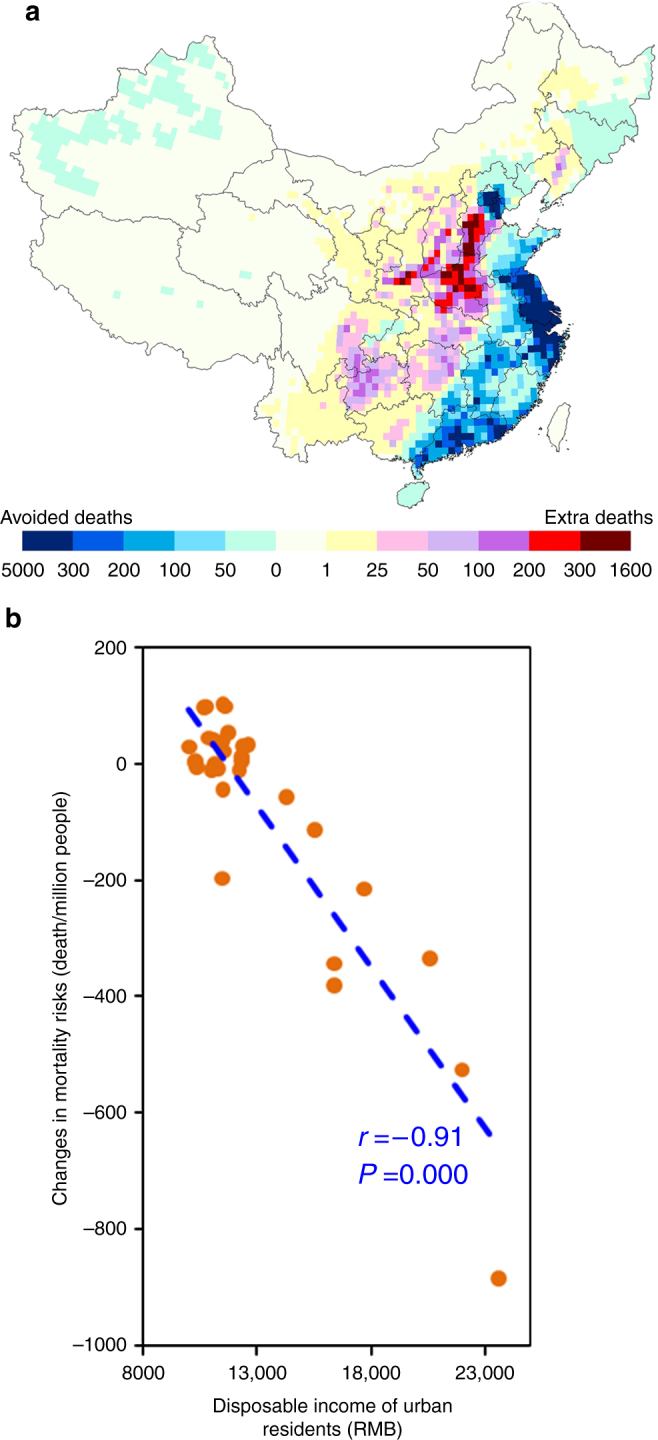
Effects of interprovincial trade on premature deaths attributable to ambient PM_2.5_ across China. **a** geographical distribution of mortality, with blue and red/lavender/yellow indicating regions of avoided deaths (a benefit) and increased deaths (a disbenefit) from interprovincial trade, respectively; **b** increased (positive values) or avoided (negative values) mortality risk (deaths per million people) of provinces by economic development level, indicated by per capita disposable income of urban residents (RMB)

In order to evaluate the effects of interprovincial trade on ambient PM_2.5_ concentrations (Supplementary Fig. 1c) and related health impacts across China (Fig. 3a), we counterfactually relocate interprovincial trade-related emissions to provinces based on consumption rather than production (scenario 3 in Supplementary Table 2). Comparison with the analogous results for the actual production-based emission distribution suggests that interprovincial trade reduced China’s general PWM-PM_2.5_ by 3.8 μg m^−3^ (6.9%), avoiding 78,500 (CI95: 59,700–96,200) deaths. This means that current interprovincial trade resulting from specialization and relocation of industries has potentially improved the resource utilization efficiency^16^, and therefore mitigated the adverse health impact attributable to ambient air pollution for China as a whole. The most developed, coastal regions and provinces gain the greatest air pollution health benefits from interprovincial trade, including Beijing (−43.2 μg m^−3^ in PWM-PM_2.5_ and 8600 avoided deaths), the Yangtze River Delta (Zhejiang: −22.1 and −16,800; Shanghai: −58.7 and −16,400; Jiangsu: −26.1 and −26,000), and the Pearl River Delta (Guangdong: −12.7 and −20,200). Exacerbation of air pollution and health burdens, on the other hand, was suffered by interior provinces, such as Hebei (+6.0 μg m^−3^ in PWM-PM_2.5_ and 3800 additional deaths), Henan (+9.3 and +9700), Guizhou (+6.2 and +3700).

Effects of interprovincial trade on mortality risks associated with air pollution are inversely related to provincial per capita wealth (Fig. 3b), revealing an important inequity between coastal and interior provinces. Stated in more stark terms, some of the gains from economic development in China’s coastal regions are likely at the expense of negative environmental and health impacts on interior regions (Fig. 3a). The relatively poor medical services in less developed interior provinces may worsen the adverse health impacts, potentially further offsetting the economic gains from interprovincial trade.

Note that mitigation of air pollution in coastal regions achieved by relocating emissions to interior provinces through interprovincial trade can be partially canceled by long-range atmospheric transport and chemistry. For instance, gaseous SO_2_ and NO_X_ are less harmful but can be transformed into sulfate and nitrate PM_2.5_ through secondary chemical processing noted above^17^, yielding longer atmospheric lifetimes and transport distances than those for primary PM_2.5_. To the extent that prevailing winds transport pollution from China’s interior provinces to coastal regions^18^, a function of seasonal meteorology, the latter can be affected by elevated PM_2.5_ from the upwind pollutant sources. These complex effects are captured in our results by nested-grid GEOS-Chem modeling of atmospheric transport and chemistry.

## Discussion

Both international and interprovincial trade have exacerbated air pollution and associated health burdens in China’s interior provinces. Relatively high emission intensities of some industries in a number of less developed interior provinces of China may be a key factor driving the large emissions embodied in both international exports and interprovincial trade in China. Targeted shifts to cleaner energy sources and modernization of technology used by related industries of those interior provinces might alleviate the air pollution associated with Chinese trade. If the emission intensities of interprovincial exports from five interior provinces (Hebei, Henan, Inner Mongolia, Shanxi, and Liaoning) were equal to levels of Shanghai, the total SO_2_ emissions embodied in China’s interprovincial trade would have been reduced by 20.0% in 2007, an amount equivalent to the emissions of Canada (2.16 Mt). But existing environmental controls, including the Action Plan for Air Pollution Prevention and Control launched in 2013 to combat increasingly serious haze events in China, still focuses primarily on the developed coastal regions where the emission intensities are relatively low. In contrast, environmental regulations are looser for interior provinces (e.g., Henan and Hebei), where the emission intensities and health burdens of air pollution are relatively high. Even as the central government is seeking to reduce national inequities through economic development of China’s interior provinces, air pollution control policies may encourage further relocation of heavy industries to those same provinces, aggravating local health burdens at the same time they improve them on a net national basis.

Our results show the complexity of regional air pollution issues, which need to be addressed by comprehensive consideration of entire production chains, atmospheric transport and chemistry, and population distributions. Since interprovincial trade between provinces generally improves the air quality in coastal provinces but degrades it in interior regions, policies should be designed to favor transfer of advanced technologies for production and emissions control from the coastal regions to the interior regions. Similarly, as Chinese industries generally emit more pollutants per unit output than their counterparts in developed countries, such domestic efforts are likely insufficient to mitigate total air pollutant emissions from a global perspective. In contrast to the global impact of greenhouse gas emissions, the health impacts of air pollution are mainly local or regional. International cooperation is critical to accelerate the diffusion of advanced technologies in manufacturing, non-fossil energy, and pollution control to and within China. Programs undertaken by multilateral development agencies (e.g., the World Bank) might provide one institutional mechanism for such technology transfer. If a global market in carbon allowances eventually emerges under the United Nations Framework Convention on Climate Change, it might also serve such aims as a co-benefit. Because fossil fuel combustion generally emits both CO_2_ and domestic air pollutants, in many cases low-cost carbon reductions in China’s interior provinces effectively sold to developed nations would also help to improve the regional air quality in China.

## Methods

### Analytical framework of this study

We consider trade between 30 Chinese provinces and 40 other countries/regions in one integrated model, evaluating the footprint of embodied emissions. Building on this approach, we further quantify the impact of both international exports and interprovincial trade on premature deaths attributable to ambient PM_2.5_ across China. Our analytical approach includes three major components (Supplementary Fig. 6). First, we track air pollutant emissions embodied in trade between Chinese provinces and other countries/regions using a global linked MRIO model, which connects China’s provincial MRIO model with the World Input−Output Database (WIOD) (http://www.wiod.org/new_site/home.htm). Second, we apply the nested-grid GEOS-Chem model (http://geos-chem.org/) to estimate the spatial change ratios of ambient PM_2.5_ concentrations induced by trade, which we then calibrate using satellite-derived PM_2.5_ concentrations to estimate trade-related PM_2.5_ exposures. Third, an integrated exposure-response (IER) model (Supplementary Fig. 7) is applied to examine the effects of trade on ambient PM_2.5_-related mortality across China.

### Evaluation of trade-embodied emissions

Calculation of the international and interprovincial trade-related emissions is based on an environmentally extended MRIO analysis of the interactions between different economic sectors and regions, multiplied by sector-specific emission intensities.

We developed a linked MRIO model (Supplementary Fig. 8) to analyze the interactions between different economic sectors and regions by integrating China’s MRIO model with the WIOD (Supplementary Tables 3–6) in this study. The key steps to create the linked MRIO model are to establish the intermediate delivery matrix (IDM) and final demand matrix (FDM) of China’s provincial goods and services exported to other countries, and those of foreign goods and services exported to individual Chinese provinces. We estimate them based on the principle that the sum of the IDM and FDM of China’s provincial goods and services exported to other countries is equal to the Chinese provincial export matrix, and the sum of those of foreign goods and services exported to individual Chinese provinces is equal to the provincial import matrix (see Supplementary Methods for the detailed process and data used for developing this linked MRIO). The linked MRIO model can be expressed as:

1 $${{\bf{X}}^*} = {\left( {{\bf{I}} - {{\bf{A}}^*}} \right)^{ - 1}}{{\bf{Y}}^*},$$

where **X** ^∗^ is the total economic output matrix; **A** ^∗^ is the matrix block of normalized matrix of intermediate coefficients in which the columns reflect the input from each sector in a region *r* required to produce one unit from each sector in region *s*; *r* and *s* represent the 70 regions (i.e., 30 Chinese provinces and 40 other countries/regions); **I** is the identity matrix; and (**I** − **A** ^∗^)^−1^ is the Leontief inverse matrix.

Sector-specific emission intensities of air pollutants are calculated as total production-based emissions divided by total monetized outputs from the respective sectors. We employed the multi-resolution emission inventory for China (MEIC)^19^ and emissions database for global atmospheric research (EDGAR)^20^ to estimate the sector-specific emissions for Chinese provinces and other countries/regions, respectively. Due to the different classification of sectors between the emission inventories and the linked MRIO model, some of the sectors are combined or divided through a mapping process as shown in Supplementary Fig. 9 and Supplementary Table 7 (see Supplementary Methods for a detailed discussion). The environmentally extended linked MRIO model can be written as:

2 $${\bf{E}}_d^* = \widehat {\bf{F}}\left[ {{{\left( {{\bf{I}} - {{\bf{A}}^*}} \right)}^{ - 1}}{{\bf{Y}}^*}} \right],$$

where **E** ^∗^ is the air pollutant emissions (e.g., SO_2_, PM_2.5_) embodied in the trade flow; **F** is a row vector of per unit output of emissions (i.e., emission intensity) for each sector in the 70 regions; $\widehat {\bf{F}}$ is the diagonalization of **F**; *d* represents the 21 sectors in the linked MRIO.

Then, the emissions generated in one region (i.e., production-based emissions) can be quantitatively decomposed into the components related to consumption activities in that region as well as in other regions (Eq. 3). The emissions related to regional consumption (i.e., consumption-based emissions) can be decomposed into the components generated within the region’s geographical boundary and those embodied in imports from outside of it, driven by the region’s consumption activities (Eq. 4).

3 $${\bf{E}}_{{\rm{p}}d}^r = {\bf{E}}_d^{rr} + \mathop {\sum}\nolimits_{\begin{array}{*{20}{c}} {s1 = 1} \\ {s1 \ne r} \end{array}}^{30} {{\bf{E}}_d^{rs1}} + \mathop {\sum}\nolimits_{\begin{array}{*{20}{c}} {s2 = 31} \\ {s2 \ne r} \end{array}}^{70} {{\bf{E}}_d^{rs2}} ,$$

4 $${\bf{E}}_{{\rm{c}}d}^r = {\bf{E}}_d^{rr} + \mathop {\sum}\nolimits_{\begin{array}{*{20}{c}} {s1 = 1} \\ {s1 \ne r} \end{array}}^{30} {{\bf{E}}_d^{s1r}} + \mathop {\sum}\nolimits_{\begin{array}{*{20}{c}} {s2 = 31} \\ {s2 \ne r} \end{array}}^{70} {{\bf{E}}_d^{s2r}} ,$$

where, ${\bf{E}}_{{\rm{p}}d}^r$ and ${\bf{E}}_{{\rm{c}}d}^r$ denote emissions associated with the products produced and consumed in a region, respectively; and *s*1 and *s*2 represent the 30 Chinese provinces and 40 other countries/regions, respectively; ${\bf{E}}_d^{rr}$ represents the emissions of products produced and consumed locally in *r*; ${\bf{E}}_d^{rs1}$ and ${\bf{E}}_d^{rs2}$ represent emissions of products produced in *r* but consumed in other Chinese provinces or other countries/regions, respectively; ${\bf{E}}_d^{s1r}$ and ${\bf{E}}_d^{s2r}$ represent emissions of product consumed in region *r* but produced in other Chinese provinces or other countries/regions, respectively.

Such MRIO-based analyses have already been widely applied to calculate embodied carbon emissions^10, 11, 21^ associated with international trade. CO_2_ emissions have also been calculated based on our linked MRIO model, and the resulting emissions embodied in trade are generally consistent with Feng et al.^7^ (Supplementary Tables 3 and 4).

### Assessment of trade’s impact on ambient PM_2.5_ pollution

We defined six scenarios to analyze the impacts of interprovincial and international trade on air pollution across China (Supplementary Table 2). The production-based emissions (i.e., MEIC and EDGAR) serve as a baseline emission scenario (Scenario 1). Scenario 2 is designed to analyze the impact of global exports from China on the emissions of all provinces, by omitting from Scenario 1 the emissions embodied in international exports from relevant sectors of each Chinese province. Scenario 3 is designed to analyze the impact of interprovincial trade on the relocation of provincial emissions, by replacing in each province the emissions embodied in interprovincial exports in Scenario 1 by those embodied in interprovincial imports. Scenarios 4, 5, and 6 omit from Scenario 1 the emissions embodied in the exports to the US, Europe, and East Asia, respectively, for each province.

We applied the GEOS-Chem model (v10-01) to simulate the spatial change ratios of PM_2.5_ concentrations attributable to individual activities (e.g., international export, interprovincial trade) across China at a resolution of 0.5° latitude × 0.667° longitude (a nominal resolution of ~ 50 km). The spatial change ratios between baseline PM_2.5_ concentrations and the five alternative simulated results are considered as the impacts of various types of trade activities on PM_2.5_ concentrations (Supplementary Fig. 1). The calculated spatial change ratios are then multiplied with satellite-retrieved PM_2.5_ concentrations to calculate the individual trade-derived ambient PM_2.5_ concentrations across China:

5 $$A = 1 - \frac{{{C_{{\rm{mnoTRE}}}}}}{{{C_{{\rm{mtotal}}}}}},$$

6 $${C_{{\rm{TRE}}}} = {C_{{\rm{sattotal}}}} \times A,$$

7 $${C_{{\rm{noTRE}}}} = {C_{{\rm{sattotal}}}} - {C_{{\rm{TRE}}}},$$

where *A* is the spatial change ratios of PM_2.5_ concentrations attributable to trade; *C*_mtotal_ represent the GEOS-Chem-modeled PM_2.5_ concentrations using emissions under Scenario 1; *C*_mnoTRE_ represent the GEOS-Chem-modeled PM_2.5_ concentrations using emissions of Scenarios 2 ~ 6, respectively; *C*_TRE_ are the ambient PM_2.5_ concentrations induced by trade; *C*_sattotal_ represent the satellite-retrieved total PM_2.5_ concentrations; and *C*_noTRE_ represent the PM_2.5_ concentrations across China if we assume no trade.

The employed satellite-retrieved data have a high spatial resolution (10 × 10 km) and have been transformed into estimates of ground-level concentrations^22^. These data have already been extensively applied in previous studies including the global burden of disease (GBD) project^23, 24^. In order to reduce errors due to insufficient samples of satellite data, the average satellite-retrieved PM_2.5_ concentrations over 3 years (2006–2008) were used to represent the annual mean concentrations for 2007. This approach helps to correct the potential model bias if the GEOS-Chem-calculated PM_2.5_ concentrations are directly used for the baseline case, and improves the spatial resolution of our final model results. More descriptions of the GEOS-Chem simulation process and evaluation against observations are provided in Supplementary Methods.

### Evaluation of the health impacts

We quantified the premature deaths attributable to trade-induced air pollution from four major causes of mortality, i.e., ischemic heart disease (IHD), chronic obstructive pulmonary disease (COPD), stroke and lung cancer (LC), according to the GBD project:

8 $$M = \mathrm{AF} \times B \times P,$$

9 $$\mathrm{AF} = \frac{{{\sum} {{p_i}\left( {\mathrm{RR}{{\left( C \right)}_i} - 1} \right)} }}{{{\sum} {{p_i}\mathrm{RR}{{\left( C \right)}_i}} }},$$

where *M* is the premature deaths from a given disease attributable to ambient air pollution; AF is the attributable fraction of trade-driven PM_2.5_ pollution; *B* is the incidence of the given health effect due to specific disease (e.g., deaths due to IHD per 1000 people), reported at the national level due to data limitations in China^1^; *P* is the exposed population; *p*_*i*_ is the proportion of population in grid cell *i*, %; and RR(﻿*C*)_*i*_ is the relative risk of grid cell *i*.

As very limited epidemiologic research on the effects of long-term exposure to PM_2.5_ has been conducted in China, the IER functions developed by Burnett et al.^25^ were employed to calculate RR(*C﻿*) for each grid cell in this study. The IER functions describe the C−R relationship throughout a wide range of ambient PM_2.5_ concentrations (including the high levels observed in China) by integrating data from cohort studies of ambient air pollution, secondhand tobacco smoke, indoor burning of solid fuels, and active smoking. The four major causes of mortality share the same health impact function with different parameters, and can be expressed as:

10 $$RR(C) = \left\{ {\begin{array}{*{20}{l}}\\ {1 + \alpha \left[ {1 - \exp \left( { - \gamma {{\left( {C - {C_0}} \right)}^\delta }} \right)} \right]} \hfill & {{\rm{for}}\,C \;  >\; {C_0}} \hfill \\ \\ 1 \hfill & {{\rm{for}}\,C \le {C_0}} \hfill \\ \end{array}} \right.,$$

where R﻿R(*C*) is the relative risk of a given PM_2.5_ concentration *C*; *C*_0_ is the counterfactual PM_2.5_ concentration below which there is no additional risk; and *α*, *γ*, and *δ* are parameters describing the overall shape of the exposure-response curve resulting from a stochastic fitting process^25^. For each health endpoint, we implemented the RR function as in Eq. 10 using parameters *C*_0_, *α*, *γ*, and *δ* derived from Monte Carlo simulations, leading to 1000 sets of exposure-response functions. Supplementary Fig. 7 shows the plots of the exposure-response curves for different diseases, and we adopted the upper and lower bounds in these plots to represent the 95% confidence intervals (CI95). Unless indicated otherwise, we based the values of premature deaths shown here on central estimates.

Since we are unable to specify where on the nonlinear IER curve a given trade-induced PM_2.5_ concentration (*C*_TRE_) occurs, we assumed an equal probability of *C*_TRE_ throughout the distribution of concentrations, and the RR(*C*_TR﻿E_) is calculated using Eq. 11. Thus, the RR(*C﻿*_TRE_) of each grid cell can be calculated and then applied to quantify the impact of trade on premature deaths attributable to PM_2.5_ exposures:

11 $$\mathrm{RR}\left( {{C_{{\rm{TRE}}}}} \right) = \frac{1}{N}\mathop {\sum}\nolimits_{i = 0}^{N - 1} {\frac{{\mathrm{RR}\left( {i\alpha + {C_{{\rm{TRE}}}}} \right)}}{{\mathrm{RR}_{i\alpha }}}} ,$$

12 $$\alpha = \frac{{{C_{{\rm{noTRE}}}}}}{{N - 1}},$$

where *N* is the sampling number, set as 1000; *i* = 0, 1, 2,…, 998, 999; and *α* is the constant increment (Eq. 12) that divides *C*_noTRE_ (Eq. 7) into equivalent intervals.

Premature deaths are calculated for each grid cell at a resolution of 10 × 10 km and are aggregated for each province. The population densities of China in 2007 at 1 × 1 km resolution developed and applied in our previous studies^6^ were re-gridded to match the 10 × 10 km resolution of the current study. It is worth noting that this research of the impact of trade on premature death attributable to ambient PM_2.5_ across China is inherently multidisciplinary. The results should be interpreted with full consideration of the uncertainties associated with different parts of the framework.

### Data availability

The data sets generated and/or analyzed during this study are available upon request from the corresponding authors.

## Electronic supplementary material

Supplementary Information
