# Supplementary material for: Trade-driven relocation of air pollution and health impacts in China
Source: Nat Commun. 2017 Sep 29;8:738. doi: 10.1038/s41467-017-00918-5 (PMC5622044; doi:10.1038/s41467-017-00918-5)
Supplement: Supplementary file 1 — Supplementary Information [file 41467_2017_918_MOESM1_ESM.pdf]

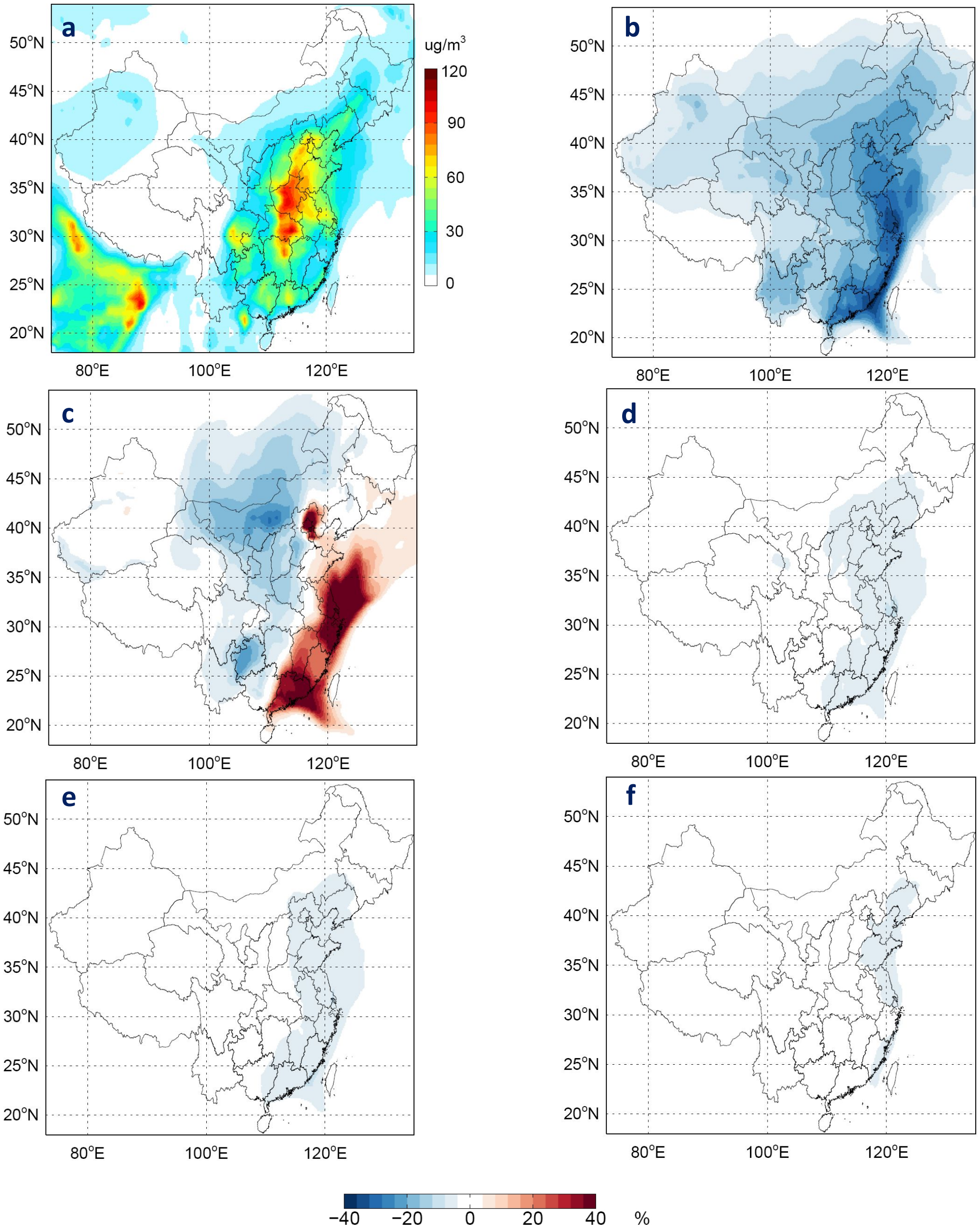

Supplementary Figure 1. Simulated impacts of trade on ambient $PM_{2.5}$ concentrations across China. We simulate ambient $PM_{2.5}$ concentrations (C_{S1}, C_{S2}, …, C_{S7}) across China by running GEOS-Chem using emissions under the six scenarios defined in section S2.3. The spatial change ratios (%) of modeled $PM_{2.5}$ concentrations of C_{S2} through C_{S6} compared to C_{S1} are considered the impacts of various types of trade on $PM_{2.5}$ concentrations across China. The baseline $PM_{2.5}$ concentrations (a), and spatial change rates of $PM_{2.5}$ concentrations from: (b) international exports from China, $(C_{S2}-C_{S1})/C_{S1}$, %; (c) interprovincial trade, $(C_{S3}-C_{S1})/C_{S1}$, %; (d) exports to the US, $(C_{S4}-C_{S1})/C_{S1}$, %; (e) exports to Europe, $(C_{S5}-C_{S1})/C_{S1}$, %; (f) exports to East Asia, $(C_{S6}-C_{S1})/C_{S1}$, %.

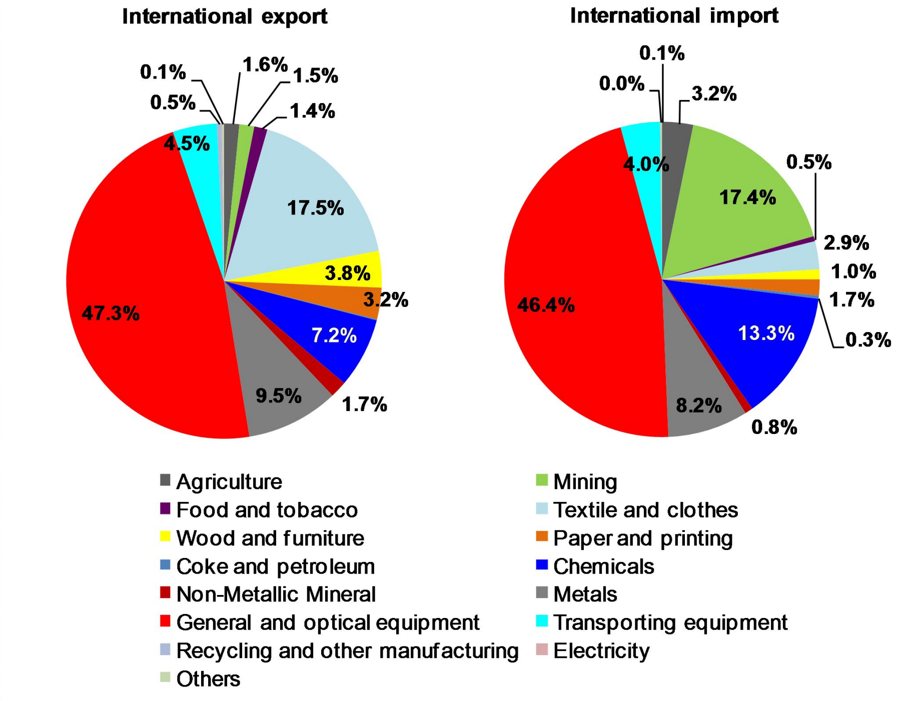

Supplementary Figure 2. The sector composition of international exports and imports of China in 2007.

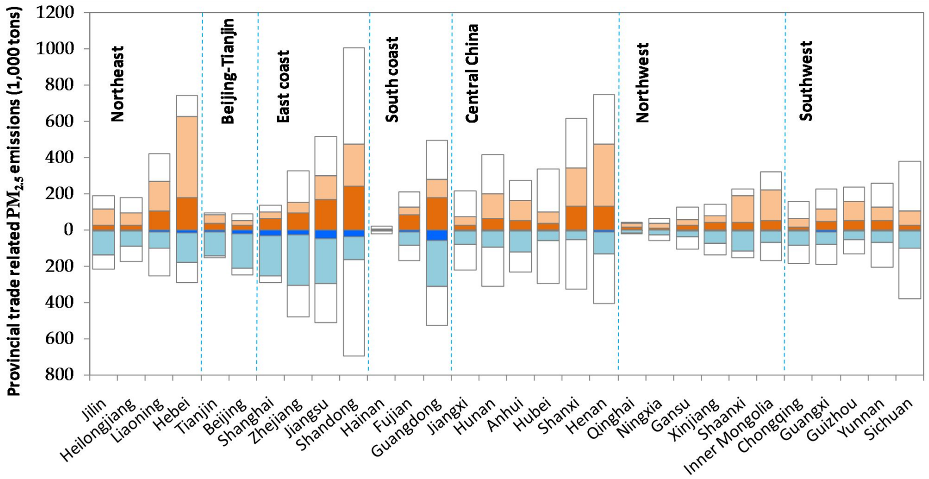
Provincial trade related $PM_{2.5}$ emissions (1,000 tons)
Northeast
Beijing-Tianjin
East coast
South coast
Central China
Northwest
Southwest
Jilin
Heilongjiang
Liaoning
Hebei
Tianjin
Beijing
Shanghai
Zhejiang
Jiangsu
Shandong
Hainan
Fujian
Guangdong
Jiangxi
Hunan
Anhui
Hubei
Shanxi
Henan
Qinghai
Ningxia
Gansu
Xinjiang
Shaanxi
Inner Mongolia
Chongqing
Guangxi
Guizhou
Yunnan
Sichuan

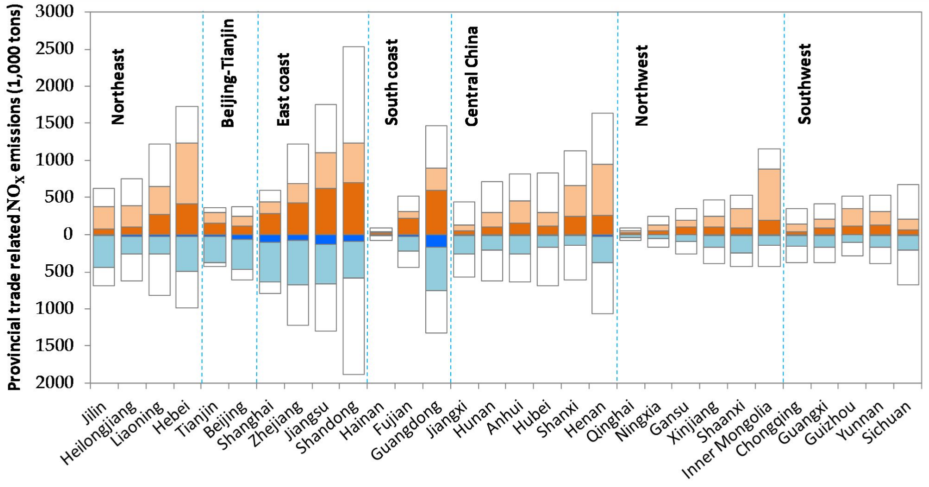
Provincial trade related NO_X emissions (1,000 tons)
Northeast
Beijing-Tianjin
East coast
South coast
Central China
Northwest
Southwest
Jilin
Heilongjiang
Liaoning
Hebei
Tianjin
Beijing
Shanghai
Zhejiang
Jiangsu
Shandong
Hainan
Fujian
Guangdong
Jiangxi
Hunan
Anhui
Hubei
Shanxi
Henan
Qinghai
Ningxia
Gansu
Xinjiang
Shaanxi
Inner Mongolia
Chongqing
Guangxi
Guizhou
Yunnan
Sichuan

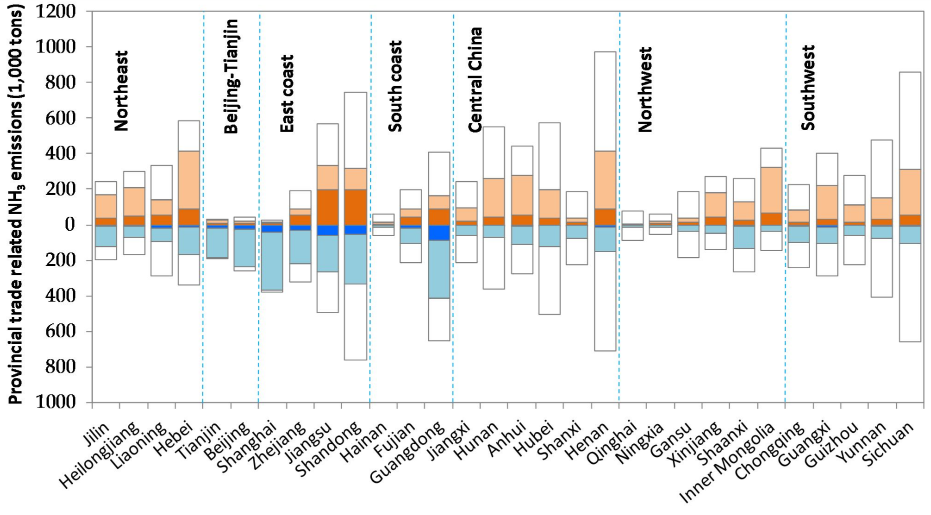
Provincial trade related NH_3 emissions (1,000 tons)
Northeast
Beijing-Tianjin
East coast
South coast
Central China
Northwest
Southwest
Jilin
Heilongjiang
Liaoning
Hebei
Tianjin
Beijing
Shanghai
Zhejiang
Jiangsu
Shandong
Hainan
Fujian
Guangdong
Jiangxi
Hunan
Anhui
Hubei
Shanxi
Henan
Qinghai
Ningxia
Gansu
Xinjiang
Shaanxi
Inner Mongolia
Chongqing
Guangxi
Guizhou
Yunnan
Sichuan

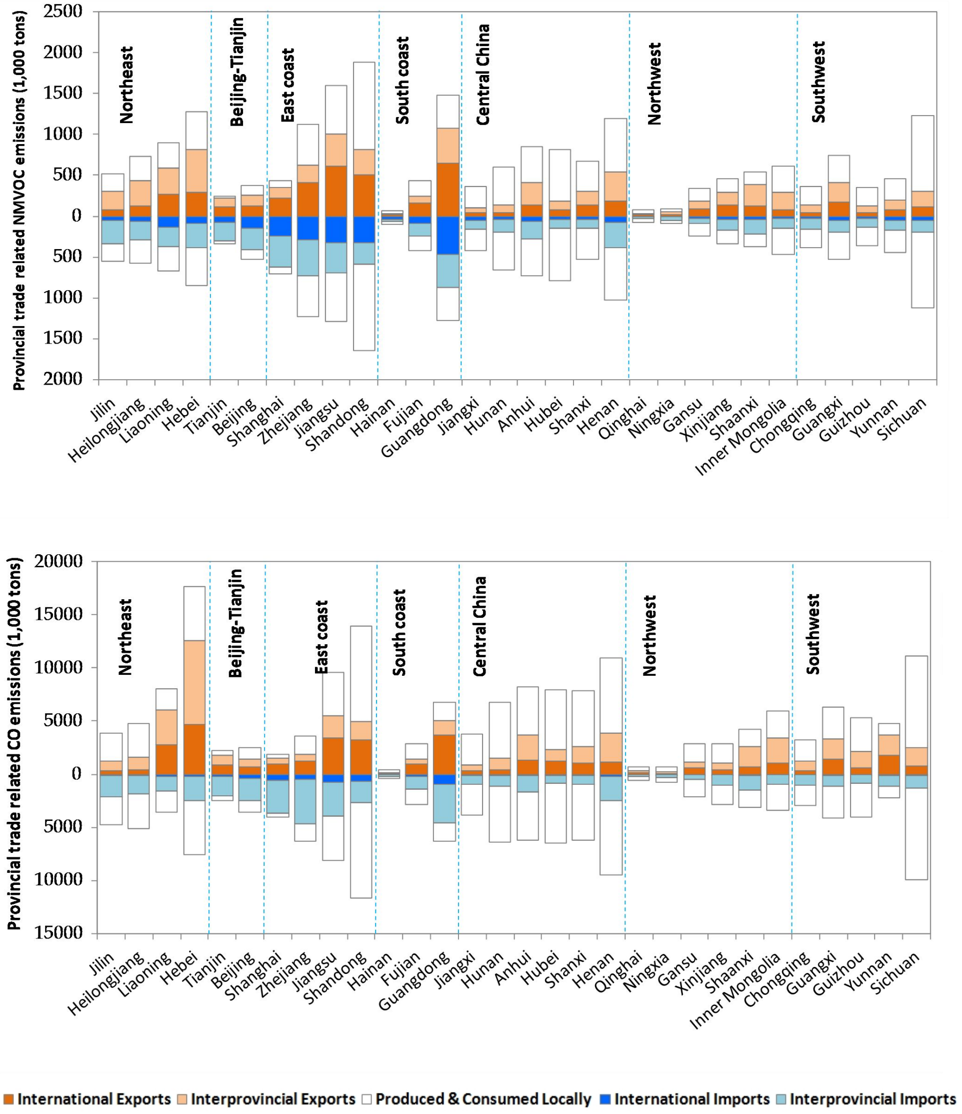

Supplementary Figure 3. Comparison of provincial air pollutant emissions from production and consumption perspectives. The production- and consumption-based emissions of primary $PM_{2.5}$, NO_X, NH_3, NMVOCs and CO by province in 2007 are illustrated on top and bottom, respectively.

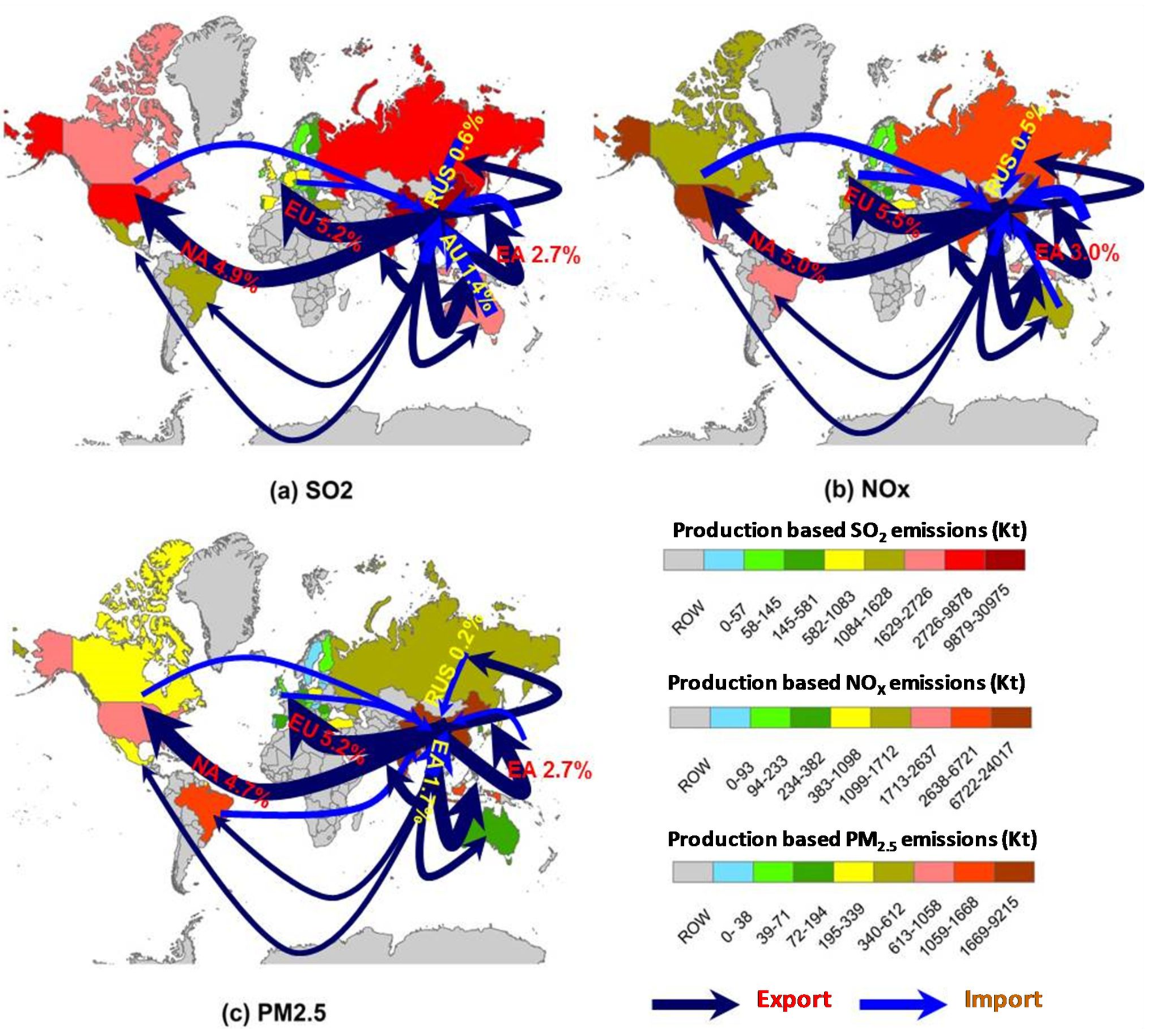

Supplementary Figure 4. Primary air pollutant emissions embodied in international trade between China and other countries or regions in 2007. (a) SO_2 emissions, 1,000 tons; (b) NOX emissions, 1,000 tons; (c) $PM_{2.5}$ emissions, 1,000 tons.

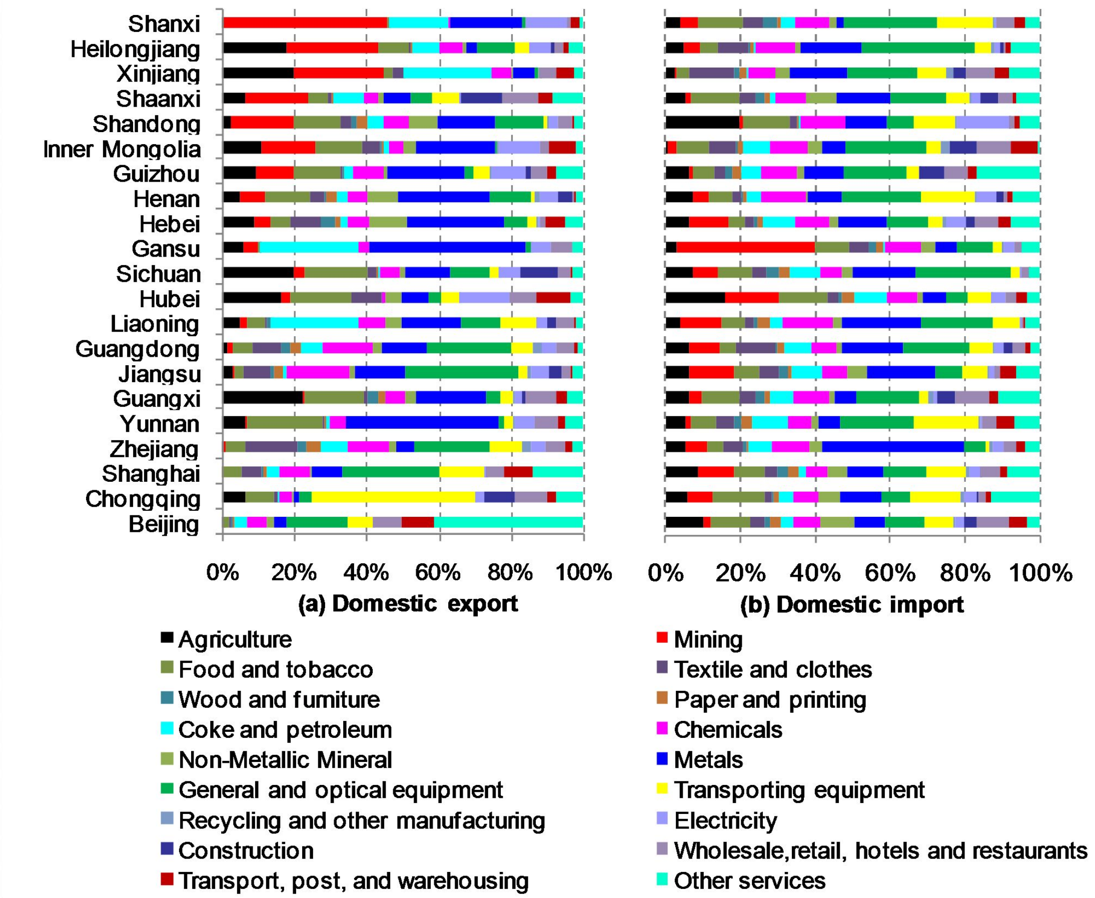

Supplementary Figure 5. The sector compositions of interprovincial exports and imports of Chinese provinces in 2007.

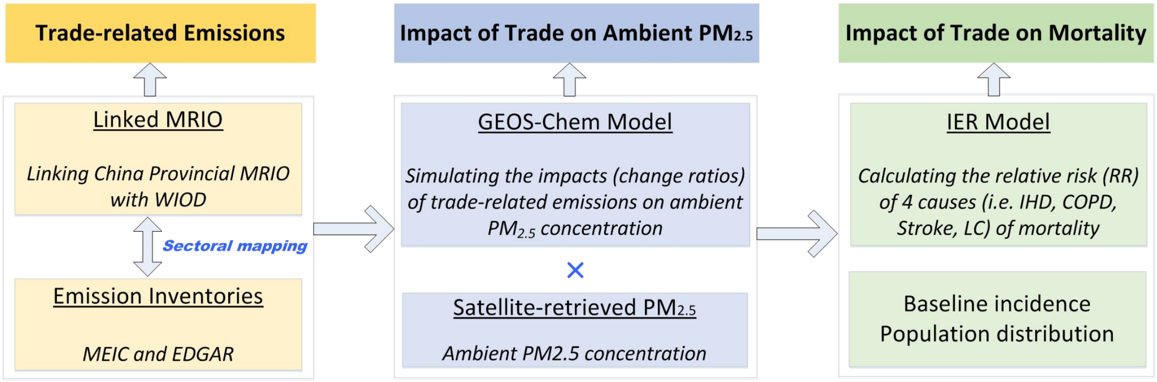

Supplementary Figure 6. Methodological framework for analyzing the effects of trade on premature death attributable to ambient $PM_{2.5}$ pollution. The framework consists of three major steps: first, we track emissions embodied in trade between Chinese provinces and other countries/regions using a global linked MRIO model, which integrates the Chinese provincial MRIO model with the World Input-Output Database (WIOD). Second, the nested-grid GEOS-Chem model is applied to estimate the spatial change ratios of ambient surface $PM_{2.5}$ concentrations, calibrated by satellite-derived $PM_{2.5}$ concentrations to estimate the impacts of trade on $PM_{2.5}$ exposures. Third, an integrated exposure-response model (IER) is then applied to examine the effects of trade on premature deaths attributable to $PM_{2.5}$ pollution across China.

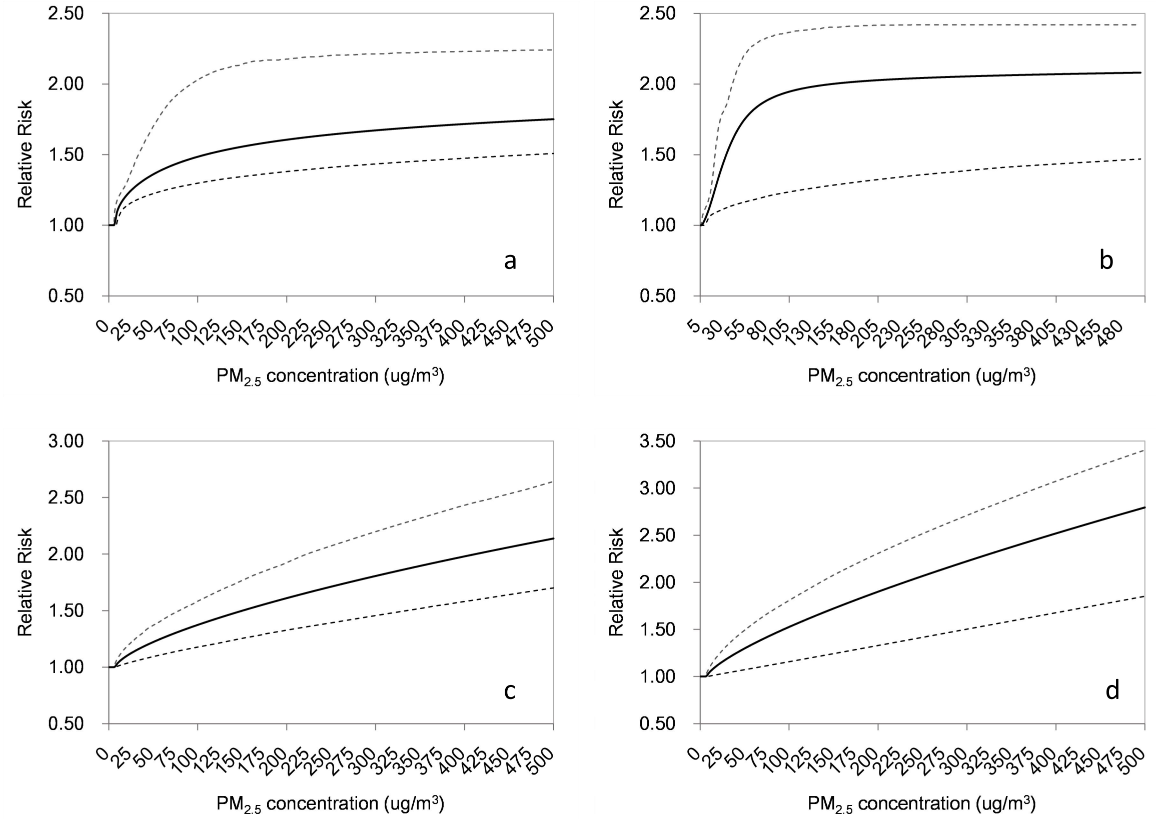

Supplementary Figure 7. The relative risk curves versus ambient $PM_{2.5}$ concentration. The mean values (solid line) and 95% confidence intervals (dashed line) of relative risks (RR) simulated using the IER model for IHD (a), stroke (b), COPD (c) and LC (d).

Supplementary Table 1. Air pollutant emissions per unit output of various sectors for China (CH), Japan (JAP) and United States (US). We aggregated the 21 sectors of the linked MRIO model (Supplementary Table 5 and 6) into 18 sectors (i.e., "textiles" and "clothes" are aggregated into "4 Textiles and clothes"; "Wholesale and retail trade" and "Hotels and restaurants" are aggregated into "16 Wholesale, retail, hotels and restaurants"; "General equipment" and "Electrical and optical equipment" are aggregated into "11 General and optical equipment" to make the result of sector analysis more clear.

ID	Sectors	SO_2 (g/dollar)			NO_x (g/dollar)			$PM_{2.5}$ (g/dollar)		
		CH	JAP	US	CH	JAP	US	CH	JAP	US
1	Agriculture	0.75	0.00 [a]	0.02	0.57	0.20	1.11	0.30	0.10	0.63
2	Mining	1.15	1.15	0.48	0.49	1.19	0.45	0.08	0.13	0.10
3	Food and tobacco	1.29	0.13	0.20	0.56	0.02	0.04	0.12	0.01	0.02
4	Textile and clothes	0.70	0.39	0.29	0.31	0.28	0.33	0.06	0.03	0.02
5	Wood and furniture	0.51	1.12	0.75	0.24	0.81	0.86	0.05	0.08	0.05
6	Paper and printing	4.27	0.13	0.20	1.03	0.02	0.04	0.23	0.01	0.02
7	Coke and petroleum	7.56	0.51	0.24	2.75	0.37	0.27	3.70	0.04	0.02
8	Chemicals	3.61	0.18	0.07	1.46	0.00 [a]	0.01	0.42	0.00 [a]	0.00 [a]
9	Non-Metallic mineral	9.14	0.00 [b]	0.00 [b]	6.56	0.00 [b]	0.00 [b]	12.48	0.09	0.09
10	Metals	2.69	0.07	0.20	1.82	0.01	0.02	1.69	0.05	0.02
11	General and optical equipment	0.19	0.14	0.17	0.10	0.10	0.19	0.02	0.01	0.01
12	Transporting equipment	0.27	0.36	0.38	0.16	0.26	0.43	0.02	0.02	0.02
13	Recycling and other manufacturing	0.42	0.64	0.00 [b]	0.23	3.02	0.00 [b]	0.04	0.25	0.00 [b]
14	Electricity	37.69	3.17	18.91	23.76	3.66	12.54	3.59	0.35	0.60
15	Construction	0.23	0.52	0.36	0.72	0.38	0.41	0.08	0.04	0.02
16	Wholesale, retail, hotels and restaurants	0.57	0.14	0.01	0.07	0.04	0.05	0.21	0.00 [a]	0.02
17	Transport, post, and warehousing	1.41	0.45	0.19	13.83	1.79	5.56	1.28	0.08	0.09
18	Other services	0.16	0.18	0.01	0.02	0.05	0.04	0.06	0.00 [a]	0.02

Note:

a. These values appear too small, for unclear reasons.

b. No data, due to different classification of these sectors in Japan and/or the U.S. compared to China.

Supplementary Table 2. Definitions of various scenarios used in this study

Scenarios	Definition	Calculation Equation
S1	Baseline scenario, production-based emissions from MEIC and EDGAR	*E_S1*
S2	Exclude emissions embodied in international exports (*E_GE*) from each sector of all Chinese provinces based on S1	*E_S2 = E_S1 - E_GE*
S3	Substitute emissions embodied in domestic inter-province exports (*E_DE*) with those embodied in interprovincial imports (*E_DI*) for each sector of all provinces in S1	*E_S3 = E_S1 - E_DE + E_DI*
S4	Exclude emissions embodied in the exports to US (*E_US*) based on S1	*E_S4 = E_S1 - E_US*
S5	Exclude emissions embodied in the exports to Europe (*E_Euro*) based on S1	*E_S5 = E_S1 - E_Euro*
S6	Exclude emissions embodied in the exports to East Asia (*E_EAsia*) based on S1	*E_S6 = E_S1 - E_EAsia*

Supplementary Table 3. Comparisons of provincial consumption-based CO_2 emissions (CE) between this study and Feng et al. (2013), Mt.

Result in this paper		Result in Feng et al. (2013)		Result in this paper		Result in Feng et al. (2013)	
Top 10 Provinces	CE	Top 10 Provinces	CE	Bottom 10 Provinces	CE	Bottom 10 Provinces	CE
Shandong	534	Shandong	541	Hainan	22	Hainan	22
Guangdong	432	Jiangsu	394	Qinghai	29	Qinghai	29
Jiangsu	388	Guangdong	392	Ningxia	52	Ningxia	47
Zhejiang	355	Zhejiang	385	Gansu	77	Gansu	86
Henan	304	Hebei	296	Guizhou	99	Guizhou	98
Hebei	254	Henan	294	Xinjiang	100	Xinjiang	102
Shanghai	232	Liaoning	239	Chongqing	117	Chongqing	108
Sichuan	232	Shanghai	238	Shaanxi	119	Yunnan	114
Hubei	213	Sichuan	230	Guangxi	120	Guangxi	116
Liaoning	206	Hubei	215	Tianjin	124	Tianjin	121

Supplementary Table 4. Comparisons of the top 10 provinces in terms of net CO_2 emissions embodied in domestic inter-province imports (Net DIE, the exceedance of emissions embodied in interprovincial imports over those in interprovincial exports) and in terms of net emissions embodied in domestic inter-province exports (Net DEE, exceedance of emissions embodied in interprovincial exports over those in interprovincial import) between this study and Feng et al. (2013), Mt.

Result in this paper		Result in Feng et al. (2013)		Result in this paper		Result in Feng et al. (2013)	
Top 10 Provinces	Net DIE	Top 10 Provinces	Net DIE	Top 10 Provinces	Net DEE	Top 10 Provinces	Net DEE
Zhejiang	133	Zhejiang	136	Inner Mongolia	159	Hebei	198
Shanghai	119	Shanghai	108	Hebei	152	Inner Mongolia	151
Guangdong	105	Beijing	94	Henan	87	Shanxi	94
Beijing	90	Guangdong	84	Liaoning	86	Henan	81
Tianjin	49	Jilin	48	Shanxi	79	Liaoning	63
Jiangxi	40	Tianjin	47	Guizhou	50	Guizhou	35
Jiangsu	38	Jiangxi	44	Heilongjiang	20	Gansu	11
Jilin	30	Chongqing	24	Yunnan	15	Hubei	11
Chongqing	28	Fujian	22	Anhui	15	Ningxia	7
Fujian	27	Jiangsu	14	Ningxia	8	Shandong	6

Supplementary Methods

The linked multi-regional input output model

Framework for the linked multi-regional input output model. We integrated the Chinese multi-regional input–output (MRIO) table for 30 provinces[1] and the World Input-Output Database (WIOD)[2-3] to extend the boundary of industrial supply chains from the provincial levels within China to the global level, which enables us to evaluate the impacts of international trade on provincial economic activities in China.

An input-output (IO) model comprises matrices of intermediate input **Z**, final demand **D**, value added **V**, total output **X**, and total input **X'**. We differentiate China (*CH*) and foreign countries (*FC*) as two regions in order to illustrate the method of linking the MRIO model and WIOD. The frameworks for the WIOD, Chinese provincial MRIO model, and the linked MRIO model are illustrated in Supplementary Fig. 8.

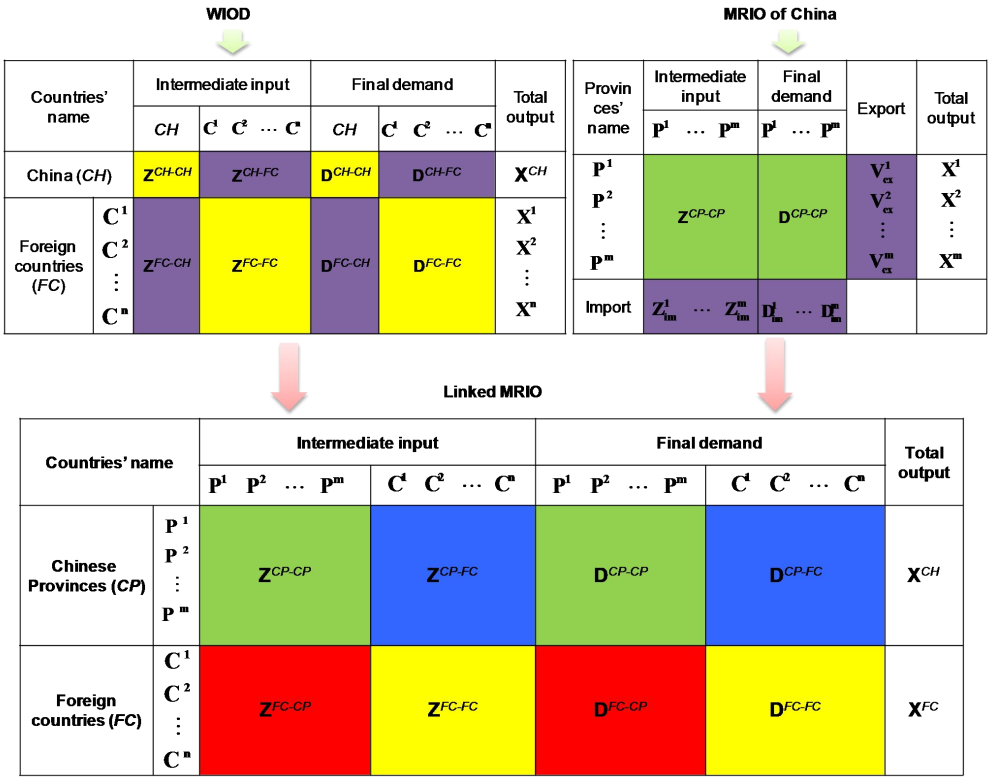

Supplementary Figure 8. Frameworks of linking the WIOD and Chinese provincial MRIO model.

Different matrices are highlighted in different colors. In the WIOD, the yellow rectangles represent intermediate delivery matrices **Z** and final demand matrices **D** within either *CH* or *FC*, respectively, and the purple rectangles represent matrices **Z** and **D** between *CH* and *FC*. The

superscripts represent the sources and destinations of goods or services traded from one place to another. For example, $\mathbf{Z}^{CH\text{-}FC}$ represents a matrix of goods and services produced in *CH* and used as intermediate inputs in *FC*. In the Chinese MRIO model, the green rectangles represent matrices **Z** and **D** for trade between Chinese provinces (*CP*). The purple rectangles represent international trade between provinces and foreign countries or regions.

In the linked MRIO model, the matrices with yellow rectangles are from the WIOD, and the matrices with green rectangles are from the Chinese MRIO model. The blue rectangles represent the intermediate delivery matrix $\mathbf{Z}^{CP\text{-}FC}$ and final demand matrix $\mathbf{D}^{CP\text{-}FC}$ of China's provincial goods and services exported to other countries, and the red rectangles represent the intermediate delivery matrix $\mathbf{Z}^{FC\text{-}CP}$ and final demand matrix $\mathbf{D}^{FC\text{-}CP}$ of foreign goods and services exported to individual Chinese provinces. Both parts should be recalculated in the linked MRIO model. Theoretically, the sum of $\mathbf{Z}^{CP\text{-}FC}$ and $\mathbf{D}^{CP\text{-}FC}$ is equal to the Chinese provincial export matrix, and the sum of $\mathbf{Z}^{FC\text{-}CP}$ and $\mathbf{D}^{FC\text{-}CP}$ is equal to the Chinese provincial import matrix. The key steps of the linked MRIO model are to estimate the matrices $\mathbf{Z}^{CP\text{-}FC}$, $\mathbf{Z}^{FC\text{-}CP}$, $\mathbf{D}^{CP\text{-}FC}$, and $\mathbf{D}^{FC\text{-}CP}$. The linking methods for trade of goods and services are different because of the different data sources.

Compilation of trade data for goods. We collected the trade data for goods from the customs statistics database in DRCNET[4], of which the commodities categories are based on the Standard International Trade Classification (SITC). We matched the sectoral trade data for goods according to the 4-digit harmonized system codes (HS code) to China's National Economic Industry Classification (GB/T 4754-2011) (Supplementary Table 5). Then, according to the Chinese 2007 Input-Output Table Establishment Method, we removed the processing and assembling trade of 30 provinces and other countries and then adjusted the charges for processing and assembling trade due to the international trade gap between the Chinese IO table and custom trade data[5]. The provincial sectoral import and export matrices are expressed as $\mathbf{V_{Im}}$ and $\mathbf{V_{Ex}}$, respectively:

$$\mathbf{V_{Im}} = \begin{pmatrix} \mathbf{v}^{1} & \cdots & \mathbf{v}^{i} & \cdots & \mathbf{v}^{m} \end{pmatrix}_{\mathbf{n \times m}} \tag{1}$$

$$\mathbf{V_{Ex}} = \begin{pmatrix} \mathbf{v}^{1} & \cdots & \mathbf{v}^{2} & \cdots & \mathbf{v}^{n} \end{pmatrix}_{\mathbf{m \times n}} \tag{2}$$

where, $\mathbf{v}^{m}$ is a column vector of sectoral import trade with other countries in province *m*, and $\mathbf{v}^{n}$ is a column vector of provincial sectoral export trade to a country *n*.

We assumed that the roles of imported goods are similar to those of local goods in China's provinces, both of which are used to satisfy local industrial production processes or consumption directly as final demand. For example, the iron ore imported from Australia is mainly used as raw material in Chinese steel production, which is similar to the iron ore produced in China[6]. Therefore, we disassembled the provincial import trade into intermediate input and final demand of each province at the sectoral level, based on the ratios of intermediate input and final demand in the Chinese MRIO model of 30 provinces. Here, we introduced the intermediate input coefficient and allocation matrices $\mathbf{H_{Im}}$ and $\mathbf{V_{Iminter}}^{FC\text{-}CP}$ and the

final demand coefficient and allocation matrices $\mathbf{W_{Im}}$ and $\mathbf{V_{Imfd}}^{FC\text{-}CP}$, respectively. The intermediate input and final demand matrices of provincial import can be expressed as:

$$\mathbf{V_{Iminter}}^{\mathbf{FC-CP}} = (\mathbf{V_{Im}})_{\mathbf{n\times m}} .\times (\mathbf{H_{Im}})_{\mathbf{n\times m}} \tag{3}$$

$$\mathbf{V_{Im\,fd}}^{\mathbf{FC-CP}} = (\mathbf{V_{Im}})_{\mathbf{n\times m}} .\times (\mathbf{W_{Im}})_{\mathbf{n\times m}} \tag{4}$$

Similarly, the exported goods from individual provinces were also disassembled into intermediate input and final demand based on the ratios of intermediate demand and final demand of different countries in the WIOD. Here, we introduce the intermediate input coefficient and allocation matrices $\mathbf{H_{Ex}}$ and $\mathbf{V_{Exinter}}^{CP\text{-}FC}$ and the final demand coefficient and allocation matrices $\mathbf{W_{Ex}}$ and $\mathbf{V_{Exfd}}^{FC\text{-}CP}$, respectively. The intermediate input and final demand matrices of provincial export can be expressed as:

$$\mathbf{V_{Ex\,inter}}^{\mathbf{CP-FC}} = (\mathbf{V_{Ex}})_{\mathbf{m\times n}} .\times (\mathbf{H_{Ex}})_{\mathbf{m\times n}} \tag{5}$$

$$\mathbf{V_{Exfd}}^{\mathbf{CP-FC}} = (\mathbf{V_{Ex}})_{\mathbf{m\times n}} .\times (\mathbf{W_{Ex}})_{\mathbf{m\times n}} \tag{6}$$

The intermediate input structure (i.e., production structure) and final demand structure of the sectoral import goods to the 30 provinces are assumed to be the same as the domestic proportion in each province in China. Therefore, we can use the “equal proportion disassembly” method, using the production structure and final demand structure of each province in the Chinese MRIO model to allocate the provincial sectoral import intermediate inputs and final demand sections to local production structures and final demand structures. Here, we introduced the production structure allocation coefficient matrix $\mathbf{P_{Im}}$ and the final demand structure allocation coefficient matrix $\mathbf{Q_{Im}}$ for Chinese provincial imports. The production structure matrix $\mathbf{Z}^{FC\text{-}CP}$ and final demand matrix $\mathbf{D}^{FC\text{-}CP}$ of provincial imports can be expressed as:

$$\mathbf{Z}^{\mathbf{FC-CP}} = (\mathbf{V_{Iminter}}^{\mathbf{FC-CP}})_{\mathbf{n\times m}} .\times (\mathbf{P_{Im}})_{\mathbf{n\times m}} \tag{7}$$

$$\mathbf{D}^{\mathbf{FC-CP}} = (\mathbf{V_{Imfd}}^{\mathbf{FC-CP}})_{\mathbf{n\times m}} .\times (\mathbf{Q_{Im}})_{\mathbf{n\times m}} \tag{8}$$

Similarly, we assumed further that the allocation of other countries’ intermediate input and final demand goods imported from China’s provinces should be similar to their local production structure and final demand structure. Therefore, we adopted each country’s production structure and final demand structure in the WIOD to disassemble the intermediate input and final demand goods from the individual Chinese provinces. Here, we introduce the production structure allocation coefficient matrix $\mathbf{P_{Ex}}$ and the final demand structure allocation coefficient matrix $\mathbf{Q_{Ex}}$ of provincial exports. The production structure matrix $\mathbf{Z}^{CP\text{-}FC}$ and final demand matrix $\mathbf{D}^{CP\text{-}FC}$ for provincial exports can be expressed as:

$$\mathbf{Z}^{\mathbf{CP-FC}} = (\mathbf{V_{Exinter}}^{\mathbf{CP-FC}})_{\mathbf{m\times n}} .\times (\mathbf{P_{Ex}})_{\mathbf{m\times n}} \tag{9}$$

$$\mathbf{D}^{\mathbf{CP-FC}} = (\mathbf{V}_{\mathbf{Exfd}}{}^{\mathbf{CP-FC}})_{\mathbf{m\times n}} .\times (\mathbf{Q}_{\mathbf{Ex}})_{\mathbf{m\times n}} \tag{10}$$

Compilation of trade data for construction and services. The international trade data for Chinese construction and services sectors are derived primarily from the Balance Table of International Payments. As there are no open official statistics of such data at the provincial level, we adopted the equal-proportion method to evaluate the provincial trade. We assumed that international imports in the construction and services sectors of each individual province have the same structures in terms of intermediate input and final demand as the domestically supplied sectors. Therefore, we disaggregated international imports for the Chinese construction and services sectors in the WIOD into 30 provinces according to the proportions of the export and import values of individual provinces in the Chinese provincial MRIO model (Supplementary Table 6).

Description of the linked MRIO model. The Chinese provincial MRIO model of 2007[1] include 30 provinces (excluding Tibet, Taiwan, Hong Kong, and Macao) and has been recently applied in several studies of regional trade-related environmental questions[7-9]. The model endogenously calculates not only the local provincial output but also the output resulting from interprovincial trade in intermediate products.

The WIOD describes trade patterns, production, consumption, and intermediate use of commodities and services among 27 European countries and 13 other major countries in the world and treats the rest of the world (ROW) as one region[3]. Therefore, our linked MRIO model involves 70 regions in total. Based on the WIOD and the Chinese provincial MRIO model, we integrated all of the trade data for goods and services into 21 sectors in the linked MRIO model, of which 16 sectors are categorized as trade in goods (Supplementary Table 5), one as construction, and the remaining four as trade in services (Supplementary Table 6). The linked MRIO model can be expressed as:

$$\mathbf{X}^* = \left(\mathbf{I} - \mathbf{A}^*\right)^{-1} \mathbf{Y}^* \tag{11}$$

where the technical coefficient $\mathbf{A}^*$ and final demand $\mathbf{Y}^*$ are:

$$\mathbf{A}^* = \begin{pmatrix} \mathbf{A}^{11} & \cdots & \mathbf{A}^{1m} & \mathbf{A}^{1(m+1)} & \cdots & \mathbf{A}^{1(m+n)} \\ \vdots & \ddots & \vdots & \vdots & \ddots & \vdots \\ \mathbf{A}^{m1} & \cdots & \mathbf{A}^{mm} & \mathbf{A}^{m(m+1)} & \cdots & \mathbf{A}^{m(m+n)} \\ \mathbf{A}^{(m+1)1} & \cdots & \mathbf{A}^{(m+1)m} & \mathbf{A}^{(m+1)(m+1)} & \cdots & \mathbf{A}^{(m+1)(m+n)} \\ \vdots & \ddots & \vdots & \vdots & \ddots & \vdots \\ \mathbf{A}^{(m+n)1} & \cdots & \mathbf{A}^{(m+n)m} & \mathbf{A}^{(m+n)(m+1)} & \cdots & \mathbf{A}^{(m+n)(m+n)} \end{pmatrix}, \quad \mathbf{Y}^* = \begin{pmatrix} \mathbf{Y}^{11} & \cdots & \mathbf{Y}^{1m} & \mathbf{Y}^{1(m+1)} & \cdots & \mathbf{Y}^{1(m+n)} \\ \vdots & \ddots & \vdots & \vdots & \ddots & \vdots \\ \mathbf{Y}^{m1} & \cdots & \mathbf{Y}^{mm} & \mathbf{Y}^{m(m+1)} & \cdots & \mathbf{Y}^{m(m+n)} \\ \mathbf{Y}^{(m+1)1} & \cdots & \mathbf{Y}^{(m+1)m} & \mathbf{Y}^{(m+1)(m+1)} & \cdots & \mathbf{Y}^{(m+1)(m+n)} \\ \vdots & \ddots & \vdots & \vdots & \ddots & \vdots \\ \mathbf{Y}^{(m+n)1} & \cdots & \mathbf{Y}^{(m+n)m} & \mathbf{Y}^{(m+n)(m+1)} & \cdots & \mathbf{Y}^{(m+n)(m+n)} \end{pmatrix}$$

where $\mathbf{A}^{rs}$ is a 21 × 21 submatrix of $\mathbf{A}^*$, representing the technical coefficients of region *s* from region *r*, which can be calculated by dividing the total input row vector $(\mathbf{X}_{\mathbf{input}})^s$ by the intermediate input matrix $\mathbf{Z}^{rs}$. $\mathbf{Y}^{rs}$ is a 21 × 5 submatrix of $\mathbf{Y}^*$, representing the final demand of

region *s* from region *r*. The total input matrix $\mathbf{X}_{\mathbf{input}}$ is the transpose of matrix $\mathbf{X}^*$ (total output), which can be described as:

$$\mathbf{X}_{\mathbf{input}} = \left(\mathbf{X}^*\right)' \tag{12}$$

As the consumption structure is distant from the objective of this paper, we had not specifically discussed the investment-related emissions although we included them in the consumption-based emissions as a whole. Investment is described as being part of final demand in the IO table and accounts for a big share of the air pollutant emissions in China. According to our previous study[10], for example, investment accounted for nearly 50% of the total consumption-based SO_2 emissions in China, much higher than some developed countries (e.g. 23% in USA, 36% in Japan).

A previous study[7] calculated the sectoral international imports and exports of Chinese provinces by disassembling provincial international trade data according to the sectoral proportion of international trade at the national level. However, the availability of natural resources, economic development, industrial structure, and level of technological advance are significantly different among Chinese provinces, and thus international trade features are distinct in various provinces. The assumption of identical sectoral shares of international trade in various provinces will bias regional differences in international trade. To avoid such bias, we have accessed to the real trade matrix of Chinese provinces to other countries at the sector level[4] when developing our linked MRIO model.

We adopted the WIOD instead of the Global Trade Analysis Project (GTAP) because the former provides more reliable information in the following respects. The original data of the WIOD originate from supply and use tables (SUTs) of countries, while those of the GTAP are obtained from their IO tables. The SUTs provide more detail on local industry, trade, production, socioeconomic conditions, environment, and other factors compared with IO tables. And, some IO tables are even compiled from the SUTs. In addition, the WIOD consists of full time-series data, while the GTAP only has data for specific years[2].

The raw data used in linking the Chinese provincial MRIO model with WIOD are derived primarily from statistics from official sources (e.g., statistical yearbooks and customs data), authoritative research centers, and peer-reviewed materials. The individual province IO tables themselves have inherent uncertainties. For example, the quality of IO tables in developed provinces (e.g., Beijing and Shanghai) tends to be better developed than those in less developed regions[11]. The data decomposition, transformation, and deduction processes used to compile the Chinese provincial MRIO model comprising 30 provinces in 2007[1] introduced additional uncertainties. Different statistical methods used to collect these raw data can also introduce uncertainty. For example, data on goods trade from customs underwent some revisions to match the data needs of the MRIO model, which increased the uncertainty of the international trade data. However, national and local official statistics are the most reliable data sources available in research on climate change and macro-environmental policy[12].

It should be noted that detailed data at a smaller scale usually have higher uncertainties compared to those at larger scales[13-14]. Therefore, uncertainties were introduced during the process of integrating, matching, and decomposing international trade data at the sectoral level when we linked the Chinese provincial MRIO model with WIOD. Fortunately, the uncertainty of small-scale data in IO analysis has less effect on the final result because the error can be largely counteracted by adding or multiplying the small-scale data using Monte Carlo methods in IO analysis[14, 15-16]. Therefore, the analysis of trade-related health impacts at China's macro provincial level, based on our linked MRIO model, is expected to be comparatively reliable.

Supplementary Table 5. Mapping goods sectors between the WIOD, Chinese provincial MRIO model, and customs data.

Linked MRIO model	WIOD	China's provincial MRIO model	Commodities category with HS code
1. Agriculture	Agriculture, hunting, forestry and fishing	Agriculture, hunting, forestry and fishing	01-05 Animal & animal products (0101-0511)<br>06-14 Vegetable products (0601-1404)<br>15 Animal or vegetable fats, oils & waxes (1501-1522)<br>99 Cotton (9952)
2. Mining	Mining and quarrying	Mining of non-metal ores and other ores<br>Mining of metal ores<br>Mining and washing of coal<br>Extraction of petroleum and natural gas	25 Salt, sulphur, earth & stone, lime & cement (2501-2530)<br>26 Ores slag & ash (2601-2621)<br>27 Mineral fuels, oils, waxes & bituminous sub (2701-2705)<br>25 (2709-2711, 2714)
3. Food and tobacco	Food, beverages and tobacco	Foods, beverages, and tobacco	16-24 Food stuffs (1601-2403)
4. Textiles	Textiles and textile products	Textile wearing apparel, footwear and caps	41-43 Raw hides, skins, leather, & furs (4101-4304)
5. Clothes	Leather, leather and footwear	Leather, fur, feather and related products	50-63 Textiles (5001-6310)<br>64-67 Footwear/headgear (6401-6704)
6. Wood and furniture	Wood and products of wood and cork	Timber, manufacture of wood, bamboo, rattan, palm, and straw products<br>Furniture	44-46 Wood & wood products (4401-4602)<br>94 Wood furniture and ornaments (9401-9406)
7. Paper printing	Pulp, paper, paper , printing and publishing	Paper and paper products<br>Printing, reproduction of recording media<br>Articles for culture, education and sports	47 Pulp of wood, waste & paper scrap (4701-4707)<br>48 Paper & paperboard, articles of paper pulp (4801-4823)<br>49 Printed books, newspapers, pictures, manuscripts, typescripts & plans (4901-4911)<br>92 Musical instruments, parts & accessories (9201-9209)<br>95 Toys, games & sports equipment (9503-9508)<br>96 Miscellaneous manufactured articles (9608-9612)<br>84 Nuclear reactors, boilers, machinery & mechanical appliances, computers (8440)
8. Coke and petroleum	Coke, refined petroleum and nuclear fuel	Processing of coking, petroleum, and nuclear fuel	27 (2706-2708)<br>27 (2712-2713, 2715)
9. Chemicals	Chemicals and relevant products<br>Rubber and plastics	Manufacture of raw chemical materials, medicines, chemical fibers, rubber	28-38 Chemicals & allied industries (2801-3825)<br>39-40 Plastics/rubbers (3901-4017)
10. Non-metallic mineral	Other non-metallic mineral	Non-metallic mineral products	68-70 Stone/glass (6801-7020)
11. Metals	Basic and fabricated metals	Metal smelting and pressing<br>Metal products	72-83 Metals (7201-8311)
12. General equipment	Machinery	General and special equipment	84 (8402-8468, 8473-8487)<br>90 Optical, photographic, cinematographic, measuring, checking, precision, medical or surgical instruments
13. Electrical and optical equipment	Electrical and optical equipment	Communication, computers, other electronic equipment<br>Measuring, machinery for cultural activity and office work	84-85 Machinery/electrical (8469-8472, 8501-8548)<br>90 (9001-9017, 9023-9033)<br>91 Clocks & watches & parts thereof (9101-9114)
14. Transport equipment	Transport equipment	Transport equipment	86-89 Transportation (8601-8908)
15. Other manufacturing	Other Manufacturing and recycling	Other manufacturing	96 Miscellaneous manufactured articles (9601-9607, 9613-9618)<br>97 Works of art. Collectors' pieces, antiques (9701-9706)<br>71 Pearls, stones, precious metals, imitation jewelry, coins (7101-7118)
16. Electricity	Electricity, gas and water supply	Electric power, gas, water production and supply	27 (2716)

Supplementary Table 6. Mapping construction and service sectors between the WIOD and Chinese provincial MRIO model

Linked MRIO model	WIOD	China's provincial MRIO model
Construction	Construction	Construction
Wholesale and retail trade	Sale, maintenance and repair of motor vehicles and motorcycles; retail sale of fuel<br>Wholesale trade and commission trade, except of motor vehicles and motorcycles<br>Retail trade, except of motor vehicles and motorcycles; repair of household goods	Wholesale and retail trade
Hotels and restaurants	Hotels and restaurants	Hotels and catering services
Transport, postage, and warehousing	Inland transport<br>Water transport<br>Air transport<br>Postage; telecommunications<br>Other supporting and auxiliary transport activities; activities of travel agencies	Transport, storage, and post
Other services	Financial intermediation<br>Real estate activities<br>Renting business activities<br>Public administration and defense; compulsory social security<br>Education<br>Health and social work<br>Other community, social and personal services<br>Private households with employed persons	Others

Calculation of trade related emissions

Environmentally extended linked MRIO model. The linked MRIO model is able to capture the economic interconnections among sectors and regions of the world. On the basis of this linked MRIO model, we conducted an environmentally extended input−output analysis to track the emissions embodied in China's international and interprovincial trade through the supply chain to the places where they are physically produced. Based on Supplementary equation 11, the environmentally extended linked MRIO model can be expressed as:

$$\mathbf{E}^{*}=\mathbf{F}\left(\mathbf{I}-\mathbf{A}^{*}\right)^{-1}\mathbf{Y}^{*},\quad \left(\mathbf{F}=\begin{pmatrix}\mathbf{F}^{1}\\ \mathbf{F}^{2}\\ \vdots \\ \mathbf{F}^{70}\end{pmatrix}^{\mathrm{T}},\quad \mathbf{F}^{r}=\begin{pmatrix}\mathbf{f}_{1}^{r}\\ \mathbf{f}_{2}^{r}\\ \vdots \\ \mathbf{f}_{21}^{r}\end{pmatrix}^{\mathrm{T}}\right) \tag{13}$$

$$\mathbf{f}_{j}^{r}=\frac{\mathbf{E}_{j}^{r}}{\mathbf{X}_{j}^{r}} \tag{14}$$

where **E*** is the environmental impact, which in this analysis refers to air pollutant emissions (e.g., SO_2, NOx, and $PM_{2.5}$) from anthropogenic activities; **F** is a row vector for per unit output of emissions (i.e., emission intensity) for each sector in the 30 Chinese provinces and the other 40 countries and regions; $\mathbf{f}_j^{r}$ is an element of a sub-vector $\mathbf{F}^{r}$ in **F**, which can be calculated using Supplementary equation 14; and $\mathbf{E}_j^{r}$ and $\mathbf{X}_j^{r}$ are the emissions and economic output of sector *j* in region *r*, respectively, in the environmentally extended linked MRIO model. Supplementary equation 13 can then be transformed into Supplementary equation 15:

$$\mathbf{E}_{d}^{*}=\hat{\mathbf{F}}\left[\left(\mathbf{I}-\mathbf{A}^{*}\right)^{-1}\mathbf{Y}^{*}\right]=\hat{\mathbf{F}}\mathbf{Y}_{l}$$

$$=\begin{pmatrix}\mathbf{F}^{1} & & & & \\ & \ddots & & & \\ & & \mathbf{F}^{r} & & \\ & & & \ddots & \\ & & & & \mathbf{F}^{(m+n)}\end{pmatrix}\begin{pmatrix}\mathbf{Y}_{l}^{11} & \cdots & \mathbf{Y}_{l}^{1s} & \cdots & \mathbf{Y}_{l}^{1(m+n)}\\ \vdots & \ddots & \vdots & \ddots & \vdots \\ \mathbf{Y}_{l}^{r1} & \cdots & \mathbf{Y}_{l}^{rs} & \cdots & \mathbf{Y}_{l}^{r(m+n)}\\ \vdots & \ddots & \vdots & \ddots & \vdots \\ \mathbf{Y}_{l}^{(m+n)1} & \cdots & \mathbf{Y}_{l}^{(m+n)s} & \cdots & \mathbf{Y}_{l}^{(m+n)(m+n)}\end{pmatrix} \tag{15}$$

where $\mathbf{E_d}^{*}$ is an emissions matrix of different sectors in different regions;   is the diagonalization of **F**; $\mathbf{Y_l}$ is the Leontief inverse $\mathbf{(I\text{-}A)^{-1}}$ transformation for the final demand $\mathbf{Y}^{*}$, representing the output of all sectors of all countries and regions to satisfy the global final demand $\mathbf{Y}^{*}$based on the global supply chains; and $\mathbf{Y_l}^{rs}$ is a submatrix of $\mathbf{Y_l}$, representing the output in region *r* due to the production of goods consumed in region *s*.

Sectoral emission intensity. Sectoral emissions ($\mathbf{E_j}^{r}$, in Supplementary equation 14) required in the calculation of the emissions intensity vector (**F**, in Supplementary equation 13) were derived using the Multi-Resolution Emission Inventory for China (MEIC, http://www.meicmodel.org) and Emissions Database for Global Atmospheric Research (EDGAR, http://edgar.jrc.ec.europa.eu).

Because the sector classifications of the emission inventories and our linked MRIO model differ, we conducted a mapping process to relate them to each other.

The MEIC is a unit/technology-based, bottom-up air pollutant emission inventory for China, updated from the widely used INTEX-B data set[17], which provides rich emission data from more than 700 emission sources and production categories[18]. This detailed information enables the direct allocations of most emission sources in MEIC into economic sectors according to the products or/and usage categories, such as the emission sources of power plants, industrial processes, and transportation. For the emissions from residential and service sectors and industrial boilers, data from the *China Energy Statistical Yearbook*[19] and sectoral energy consumption from the *China Economic Census Yearbook*[20] were used as proxies to divide their aggregated emissions and allocate them to economic sectors. Using this mapping process, Huo et al.[21] linked MEIC's emissions to 36 economic sectors, which have already been applied in previous studies[9-10, 22-23]. The emissions of the 36 sectors in Huo et al.[21] are further aggregated and mapped into the 21 sectors of our linked MRIO model (Supplementary Table 7).

Production-based emissions of other countries/regions were obtained from EDGAR v.4.2 to calculate their sectoral emission intensities. The EDGAR inventory includes the emissions of SO_2, NO_x, CO, total non-methane volatile organic compounds (NMVOCs), NH_3, as well as particulate matter compounds (i.e., PM_{10}, $PM_{2.5}$, BC, OC) for nearly 50 sources/sectors according to the IPCC 1996 code. Mapping the EDGAR emission sectors for other countries/regions to our linked MRIO model sectors followed a similar procedure as for China (see Supplementary Fig. 9). The major emission sources in EDGAR could be mapped to our linked MRIO model sectors according to their products or/and usage categories, such as the emission sources of public electricity and heat production, and transportation. For the emissions from other sources (the italic labeled sectors in Supplementary Fig. 9), e.g., the residential and other sectors, the corresponding sectoral energy consumptions from IEA (http://www.iea.org/statistics/) or the economic activities were used as proxies to allocate the aggregated emissions into the corresponding 21 economic sectors classified in our linked MRIO model.

Supplementary Table 7. Mapping the 36 economic sectors allocated from MEIC[21] to the 21 sectors in the linked MRIO model of this study

Sector No.	**Mapped sector emissions from MEIC**	*Sector No.*	**Mapping MEIC's emissions to the sectors in the Linked MRIO model**
1	Agriculture	1	Agriculture
2	Coal mining and processing	2	Mining
3	Crude petroleum and natural gas products		
4	Metal ore mining		
5	Non-metal mineral mining		
6	Food and tobacco	3	Food
7	Textiles	4	Textiles
8	Clothing, leather, and feather	5	Clothes
9	Sawmills and furniture	6	Wood stuffs
10	Paper products, printing and record medium reproduction	7	Pulp paper, printing, and publishing
11	Petroleum processing and coking	8	Coke, oil and nuclear fuel
12	Chemicals	9	Chemicals
13	Nonmetal mineral products	10	non-metallic
14	Metal smelting and pressing	11	Metals
15	Metal products		
16	Machinery and equipment	12	General equipment
17	Transport equipment	14	Transport equipment
18	Electric equipment and machinery	13	Electrical and optical equipment
19	Electronic and telecommunication equipment		
20	Instruments, meters, cultural and office machinery	15	Other manufacturing
21	Other manufacturing factors (artwork, etc.)		
22	Scrap and waste		
23	Electricity, steam and hot water production and supply	16	Electricity
24	Gas production and supply		
25	Water production and supply		
26	Construction	17	Construction
27	Transport and warehousing	20	Transport, postage, and warehousing
28	Post		
29	Wholesale, retail, trade	18	Wholesale and retail trade
30	Hotel and restaurants	19	Hotels and restaurants
31	Finance and insurance	21	Other services
32	Real estate		
33	Scientific research		
34	Public administration		
35	Culture, sports and entertainments		
36	Other service sectors (tourism, IT services, etc.)		

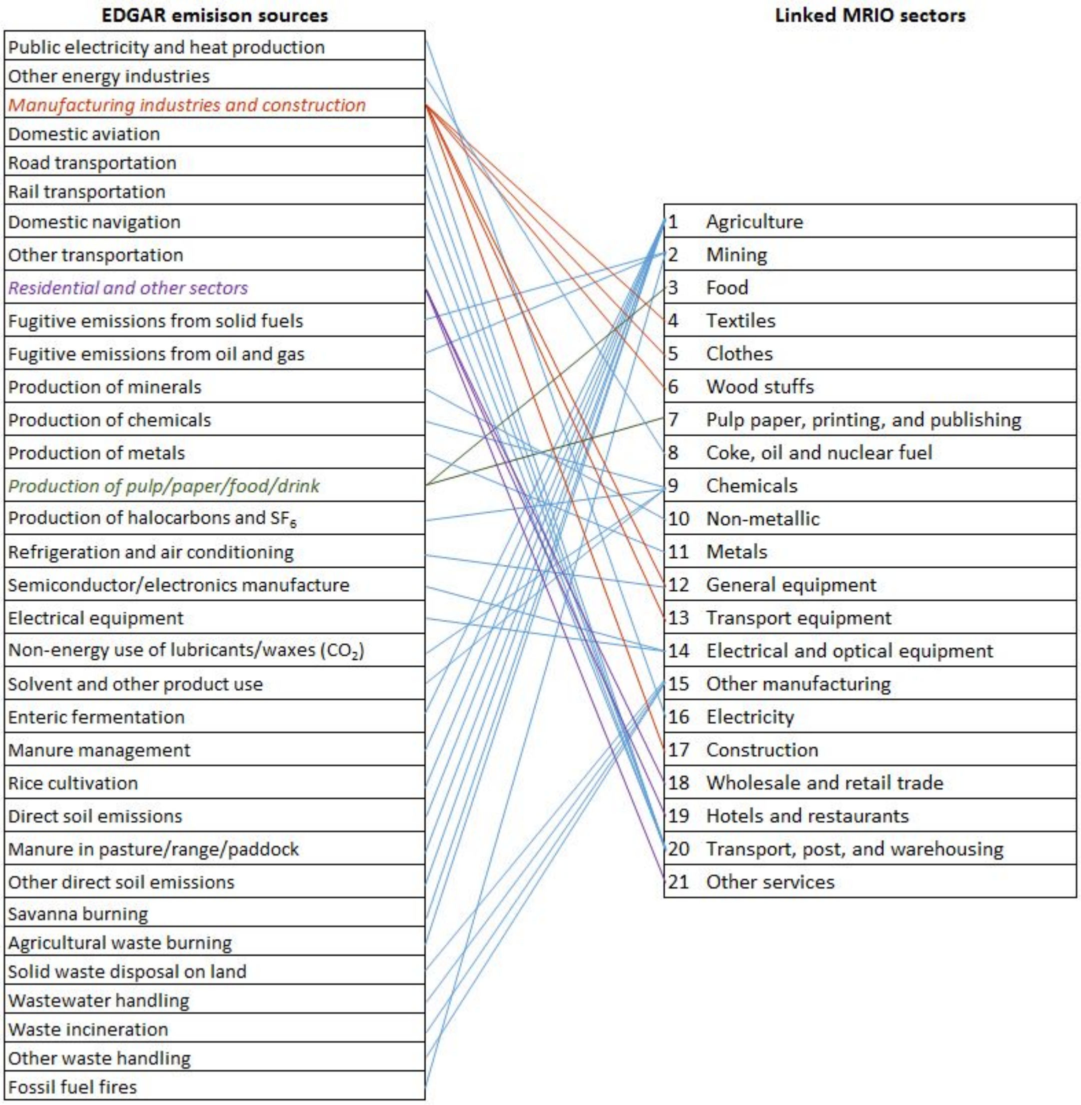

Supplementary Figure 9. Allocation of the emission sources in EDGAR to the 21 economic sectors in our linked MRIO model.

Trade-related emissions. According to Supplementary equation 15, the production-based emissions (PE) of a particular region *r* ($\mathbf{E_{pd}}^{\mathbf{r}}$) are calculated through Supplementary equation 16.

$$\mathbf{E_{pd}}^{\mathbf{r}} = \begin{pmatrix} \mathbf{F}^1 & & & & \\ & \ddots & & & \\ & & \mathbf{F}^r & & \\ & & & \ddots & \\ & & & & \mathbf{F}^{(m+n)} \end{pmatrix} \begin{pmatrix} \mathbf{0} & \cdots & \mathbf{0} & \cdots & \mathbf{0} \\ \vdots & \ddots & \vdots & \ddots & \vdots \\ \mathbf{Y_l^{r1}} & \cdots & \mathbf{Y_l^{rs}} & \cdots & \mathbf{Y_l^{r(m+n)}} \\ \vdots & \ddots & \vdots & \ddots & \vdots \\ \mathbf{0} & \cdots & \mathbf{0} & \cdots & \mathbf{0} \end{pmatrix} = \begin{pmatrix} \mathbf{0} & \cdots & \mathbf{0} & \cdots & \mathbf{0} \\ \vdots & \ddots & \vdots & \ddots & \vdots \\ \mathbf{E_d^{r1}} & \cdots & \mathbf{E_d^{rs}} & \cdots & \mathbf{E_d^{r(m+n)}} \\ \vdots & \ddots & \vdots & \ddots & \vdots \\ \mathbf{0} & \cdots & \mathbf{0} & \cdots & \mathbf{0} \end{pmatrix} \quad (16)$$

$$= \mathbf{E_d}^{\mathbf{rr}} + \sum\nolimits_{s_1=1, s \neq r}^{m} \mathbf{E_d^{rs_1}} + \sum\nolimits_{s_2=m+1, s \neq r}^{m+n} \mathbf{E_d^{rs_2}}$$

where $\mathbf{E_d}^{rr}$ denotes emissions associated with the products both produced and consumed in region *r*, and $\sum_{s=1,s\neq r}^{m+n} \mathbf{E}_d^{rs}$ is the sum of emissions of products produced in region *r* but consumed in other regions *s*. Here, s_1 signifies Chinese provinces and s_2 other countries. Therefore, if *r* is a particular province in China, $\sum_{s_1=1,s\neq r}^{m} \mathbf{E}_d^{rs_1}$ and $\sum_{s_2=m+1,s\neq r}^{m+n} \mathbf{E}_d^{rs_2}$ are the emissions associated with interprovincial export and international export of province *r*, respectively.

The consumption-based emissions (CE) of a particular region *r* ($\mathbf{E_{cd}}^r$) are calculated through Supplementary equation 17.

$$\mathbf{E_{cd}}^r = \begin{pmatrix} \mathbf{F}^1 & & & & \\ & \ddots & & & \\ & & \mathbf{F}^r & & \\ & & & \ddots & \\ & & & & \mathbf{F}^{(m+n)} \end{pmatrix} \begin{pmatrix} \mathbf{0} & \cdots & \mathbf{Y}_l^{1s} & \cdots & \mathbf{0} \\ \vdots & \ddots & \vdots & \ddots & \vdots \\ \mathbf{0} & \cdots & \mathbf{Y}_l^{rs} & \cdots & \mathbf{0} \\ \vdots & \ddots & \vdots & \ddots & \vdots \\ \mathbf{0} & \cdots & \mathbf{Y}_l^{(m+n)s} & \cdots & \mathbf{0} \end{pmatrix} = \begin{pmatrix} \mathbf{0} & \cdots & \mathbf{E}_d^{1s} & \cdots & \mathbf{0} \\ \vdots & \ddots & \vdots & \ddots & \vdots \\ \mathbf{0} & \cdots & \mathbf{E}_d^{rs} & \cdots & \mathbf{0} \\ \vdots & \ddots & \vdots & \ddots & \vdots \\ \mathbf{0} & \cdots & \mathbf{E}_d^{(m+n)s} & \cdots & \mathbf{0} \end{pmatrix} \quad (17)$$

$$= \mathbf{E}_d^{rr} + \sum_{s_1=1,s\neq r}^{m} \mathbf{E}_d^{s_1 r} + \sum_{s_2=m+1,s\neq r}^{m+n} \mathbf{E}_d^{s_2 r}$$

where $\sum_{s=1,s\neq r}^{m+n} \mathbf{E}_d^{sr}$ is the sum of emissions of product consumed in region *r* but produced in other regions *s*. Similar to $\mathbf{E_{pd}}^r$, if *r* is a particular province in China, $\sum_{s_1=1,s\neq r}^{m} \mathbf{E}_d^{s_1 r}$ and $\sum_{s_2=m+1,s\neq r}^{m+n} \mathbf{E}_d^{s_2 r}$ are the emissions associated with interprovincial imports and international imports of province *r*, respectively.

Therefore, the impact of trade (international and/or interprovincial trade) on the emissions of a specific Chinese province (and sectors) can be quantified though Supplementary equation 16and Supplementary equation 17. Here, we define 6 scenarios (Supplementary Table 2) to analyze the impacts of interprovincial and international trade on the relocation of air pollutant emissions across China.

Simulations of impact of trade on ambient $PM_{2.5}$ in China

GEOS-Chem model. The impact of trade on Chinese air quality is simulated by the nested-grid chemical transport model, GEOS-Chem (v10-01, http://geos-chem.org/). Simulations are performed at a horizontal resolution of 1/2° latitude by 2/3° longitude over the East Asian region including China. This high-resolution model domain is embedded in a global chemical transport simulation of horizontal resolution 4° latitude by 5° longitude, with the latter providing

initial and boundary conditions for all simulated chemical species. The model is driven by the meteorological data from the Goddard Earth Observing System (GEOS, version 5) of the NASA Global Modeling Assimilation Office (GMAO). It contains 47 vertical layers up to 0.01 hPa. GEOS-Chem uses the same advection algorithm as the GEOS general circulation model (http://gmao.gsfc.nasa.gov/GEOS/). Convective transport in GEOS-Chem is computed from the convective mass fluxes in the meteorological archive. Boundary layer mixing in GEOS-Chem is calculated by a non-local scheme[24]. Wet deposition by rain is considered for both water-soluble aerosols and gases, and scavenging by snow and cold/mixed precipitation is also considered for aerosols[25]. Dry deposition is calculated based on the resistance-in-series scheme for all the species, with gravitational settling for dust and coarse sea salt[26].

The simulation contains a gas phase HOx–NOx–VOC-ozone-BrOx chemistry, which considers the production and loss of ozone through reactions with HOx, NOx, VOC and BrOx[27-29]. GEOS-Chem includes detailed sulfate-nitrate-ammonium-carbonaceous-dust-sea salt aerosol chemistry, which is coupled to gas phase chemistry. The model considers the thermodynamics of inorganic aerosols and the in-cloud sulfate formation based on cloud water pH. GEOS-Chem also simulates the dust and the sea salt aerosol in different size bins. Aerosols interact with gas-phase chemistry in GEOS-Chem through the effect of aerosol extinction on photolysis rates, heterogeneous chemistry, and gas-aerosol partitioning of semi-volatile compounds.

GEOS-Chem simulations require information on the spatial distributions of emissions. The impacts of trade on emissions, however, can only be quantified at the provincial level in China through the linked the WIOD and MRIO model. Following the approach adopted by earlier studies[30-31], we assumed that trade-related emissions exhibit the same spatial distribution as total emissions specified in the MIX Asian emission inventory (http://mix.greenresource.cn), which has already integrated the MEIC as an emission inventory for China and has been applied in GEOS-Chem. In the current research, the emission inventories for Scenarios 2-6 (Supplementary Table 2) can be used as inputs for the GEOS-Chem model. The original emission inventory has a spatial resolution of 1/4° by 1/4° and was re-gridded into the GEOS-Chem grid by the HEMCO emission module[32]. Regarding species for which we have estimates from all anthropogenic sources, including SO_2, NO_X, NMVOCs, NH_3, CO and $PM_{2.5}$ (BC and OC are scaled for the same factors as $PM_{2.5}$ because of similar source contributions)[33-38], the emissions from each grid cell $E_{i,j}^{GEOS\text{-}Chem}$ are calculated based on the MIX inventory $E_{i,j}^{MIX}$ adjusted by the ratio of provincial emissions from our scenarios E_k^{MRIO} and the MIX inventory E_k^{MIX}:

$$E_{i,j}^{GEOS\text{-}Chem} = E_{i,j}^{MIX} \frac{E_k^{MRIO}}{E_k^{MIX}} \qquad (18)$$

where i and j are the grid box index in latitudinal and longitudinal directions, respectively, and k is the provincial index for this grid box.

The anthropogenic emissions outside of the East Asia domain, which are required by the coarser resolution global model, are obtained from the EDGAR global emission inventory, with various

regional patches from region-specific inventories: NEI for the US (http://www.epa.gov/ttnchie1/net/2011inventory.html), CAC for Canada (http://www.ec.gc.ca/inrp-npri), BRAVO for Mexico (http://www.epa.gov/ttnchie1/conference/ei12/mexico/bravo.pdf), and EMEP for Europe (http://www.eea.europa.eu/themes/air/emep-eea-air-pollutant-emission-inventory-guidebook).

For all non-anthropogenic emissions, such as wild fires and lightning, we used the default emission inventories recommended by the GEOS-Chem model (http://wiki.seas.harvard.edu/geos-chem/index.php/Emissions_overview). All emissions are assigned to a province based on their geographical locations, and the trans-boundary transports of emissions are simulated by GEOS-Chem.

Modelling the effect of trade on $PM_{2.5}$ pollution. In the ideal case, exposure can be estimated using a perturbation in emissions with compared to a baseline case to evaluate the impacts of emission changes on human exposure[39]. This comparison can be based on measured concentrations before and after a natural "experiment", such as the strict air-quality controls enacted for the 2008 Beijing Olympics or the Clean Air Act in the US[40-41].

Alternatively, studies can employ chemical transport models such as GEOS-Chem to simulate $PM_{2.5}$ concentrations based on different alternative emission scenarios relative to a baseline scenario to analyze the impact of policies on air pollution[39]. We also employ this sensitivity analysis to evaluate the impact of various trade activities on the ambient $PM_{2.5}$ pollution and related premature deaths across China. We simulate ambient $PM_{2.5}$ concentrations across China by running GEOS-Chem using emissions of the above-defined 6 scenarios. The spatial change ratios of modeled $PM_{2.5}$ concentrations of Scenario 2-6 compared to Scenario 1 represent the impacts of various types of trade (e.g., international exports from China, exports to the US only, interprovincial trade) on $PM_{2.5}$ concentrations across China (Supplementary Fig. 1). These spatial change ratios were then multiplied by satellite-retrieved $PM_{2.5}$ concentrations developed by van Donkelaar et al.[42] to calculate the ambient $PM_{2.5}$ concentrations influenced by various types of trade.

Similar methods have already been widely applied in earlier studies to quantify the impact of policies on air quality or public health, such as the health effects attributable to policies addressing climate change[43-44] and the health benefit of controlling emissions from power plants[45-46]. We should note that the relationship between emissions and ambient $PM_{2.5}$ concentrations is nonlinear, which means that the removal of individual emission sources does not engender a linear response in the modeled contributions to ambient $PM_{2.5}$ concentrations (i.e., the sum of modeled contributions of various sources to ambient $PM_{2.5}$ concentrations does not necessarily equal 100%). For example, Lelieveld et al.[47] evaluated the global premature deaths attributable to ambient $PM_{2.5}$ from various emission sources (e.g., power plants and transportation) and applied scaling corrections (about 10%) to ensure the contributions of various sources add up to 100% at the country level. Lin et al.[31] applied a similar method to evaluate the impact of Chinese emissions on O_3 pollution in the US, although the nonlinear

chemistry in O_3 formation[48] is even more complicated than that of secondary $PM_{2.5}$, which is dominated by local emission sources (e.g., over 60% of $PM_{2.5}$ concentrations in each Chinese province are contributed by emissions from the province itself)[49-50]. Although it is not perfect, in this sense, such methodology offers an effective means for the purpose of our analysis.

Model evaluation and uncertainties. The GEOS-Chem model adopted in this analysis has been widely used for studying air quality of different regions across the world including North America[36, 51-54] Europe[55-58], and Asia[59-63]. Detailed model performance has been extensively evaluated against observed data obtained from satellite retrievals, ground-based observation sites and networks, and aircraft campaigns[60, 64-68]. To correct any potential model bias caused by the relative coarse resolution, imperfect meteorological data, or incomplete atmospheric chemical reaction schemes, we used the modeled relative changes in concentrations (Supplementary Fig. 1) of different scenarios (as defined in Supplementary Table 2) and then applied them to surface concentrations derived from satellite observations.

As satellites only measure the column density of aerosols through retrieval of aerosol optical depth (AOD), van Donkelaar et al.[42, 69] applied GEOS-Chem-modeled aerosol vertical profiles and vertical profile information from the CALIOP (Cloud-Aerosol Lidar with Orthogonal Polarization) satellite instrument to allocate the retrieved AOD to near-ground concentrations. According to Boys et al.[70] and van Donkelaar et al.[42], the uncertainty in satellite-derived $PM_{2.5}$ decreases with an increase in sampling days, and annual mean $PM_{2.5}$ satellite retrievals (1999-2011) are estimated by using the 3-year moving average from 1998 to 2012. The resultant ground-level $PM_{2.5}$ estimates are highly consistent (R^2=0.81) with $PM_{2.5}$ concentrations from in-situ surface monitors (the Figure 5 in Donkelaar et al.[69]). This satellite-derived $PM_{2.5}$ concentration dataset[42] has been applied broadly in the literature, notably including the Global Burden of Disease (GBD) assessments[71], to represent the spatiotemporal distribution of $PM_{2.5}$ exposures globally.

Evaluation of the impact of trade on public health in China

Background of the health impact assessment. The air pollution to which individuals are exposed is multifaceted, typically including numerous individual gaseous compounds and particles of complex physicochemical composition[72]. Accordingly, indicator pollutants are often used to assess exposures for risk assessment and epidemiologic analysis. Evaluation of adverse health impacts associated with elevated ambient $PM_{2.5}$ concentrations has been supported by extensive epidemiological literature[73-75], and $PM_{2.5}$ is the most robust indicator of adverse impacts (e.g., mortality) of long-term exposure to air pollution in epidemiologic cohort studies[76].

Epidemiological associations between elevated ozone concentrations and premature mortality have also been observed, which are independent of associations between $PM_{2.5}$ and mortality[77-78]. Estimates of the global burden of disease attributable to outdoor air pollution can be

enhanced by the inclusion of ozone in addition to $PM_{2.5}$, as in Lelieveld et al.[47] and the GBD[71]. However, the premature deaths attributable to O_3 exposure are far fewer, around 5% globally compared to that of $PM_{2.5}$ in 2010[47, 71]. Relatively scarce O_3 monitoring data in China make current evaluation of large-scale health impacts attributable to O_3 mainly dependent on atmospheric chemistry models (e.g., TM5, CEOS-Chem, EMAC, or CMAQ), which introduce large uncertainties due to the more complicated chemical reactions of O_3 production compared to secondary $PM_{2.5}$[48].

For this reason, we focused on ambient $PM_{2.5}$ in this study and applied an integrated exposure-response (IER) model[79] to evaluate the impact of trade on premature deaths attributable to air pollution. The IER model is a mass-based concentration-response (C-R) model that integrates the relative risks (RR) of death from a variety of illnesses, including ischemic heart disease (IHD), cerebrovascular disease (stroke), chronic obstructive pulmonary disease (COPD) and lung cancer (LC), attributable to exposure to $PM_{2.5}$ over diverse concentrations (i.e., ambient air pollution, active smoking, secondhand smoking, and indoor burning of solid fuels). It thus covers the global exposure range of $PM_{2.5}$, including the high concentrations of countries like China and India. The IER model has already been widely applied, including in the GBD, to evaluate the health impacts attributable to ambient $PM_{2.5}$ in various countries. Burnett et al.[79] compared the RRs observed in the limited Chinese cohort and those predicted by the IER model. The similarity for different health endpoints (e.g., IHD, Stroke, LC) suggests that the IER model can yield reasonable predictions of the change of risk over the range of concentrations that prevail in China.

Uncertainty and limitations. It is known that analysis of the health impacts of air pollution generally has large uncertainties[80-81]. Although we have used state-of-the-art methods to estimate the impact of trade on mortality attributable to ambient $PM_{2.5}$ pollution in China, there are a number of assumptions and limitations inherent in our estimates. First, the IER model itself has uncertainty. Besides the issues such as the shape of the concentration–response functions and the possible existence and specific levels of C-R thresholds, there is considerable evidence that the chemical composition, size distribution, and sources of $PM_{2.5}$ may influence the health effects[82]. However, the relative toxicity of various constituents of ambient $PM_{2.5}$ has not been well established[47], and definitive estimates for various chemical forms or sources would go beyond the capacity of current scientific evidence required for accurate determination[83]. Therefore, current cohort epidemiologic results for adverse (e.g., mortality) impacts are still based on long-term exposure to $PM_{2.5}$ total mass. Second, uncertainty of trade-related emissions results from that of emission inventories (i.e., MEIC and EDGAR) and the economic input-output analysis. For example, the uncertainties of the MEIC were estimated to be 12% for SO_2, 31% for NO_X, 68% for NWVOC, and 107% for primary $PM_{2.5}$[17, 84], while the uncertainty of input-out analysis might be less than 50%[31]. Third, GEOS-Chem simulations are unavoidably affected by errors in meteorological inputs and imperfect representation of tropospheric chemistry. The importance of GEOS-Chem model uncertainties, however, might be substantially reduced because we focus on change ratios between the modeled concentrations of the

baseline and alternative scenarios (Supplementary Table 2), not the absolute concentration levels simulated by GEOS-Chem.

Epidemiologic studies suggest logarithmic relationships between ambient $PM_{2.5}$ concentrations and RR[79, 81], and uncertainties in the C–R functions linking ambient $PM_{2.5}$ concentrations with mortality are particularly important for health impact assessment[80]. Therefore, we evaluated the uncertainties of the premature mortality analysis associated with the C-R function for $PM_{2.5}$ based on the methods of the GBD project[47, 71], and emphasize that the 95% confidence intervals of premature death described in this study reflect only the statistical uncertainty of the RR calculated through the IER model.

Supplementary References

1. Liu, W., Chen, J. & Tang, Z. China's 30 provinces interprovincial input-output model theory and practice in 2007. China Statistics Press, Beijing (2012).
2. Timmer, M. P. The world input-output database (WIOD): contents, sources and methods. WIOD Working Paper Number 10 (2012).
3. Timmer, M. P., Dietzenbacher, E., Los, B., Stehrer, R. & de Vries, G.J. An illustrated user guide to the world input-output database: the case of global automotive production. *Rev. Int. Econ.* **23,** 575−605 (2015).
4. DRCNET. DRCNET statistical database system, available at: http://edu-data.drcnet.com.cn/web/, (last access: 16 January 2015), (2007).
5. NEAD (Economic Accounting Department of National Bureau of Statistics of China), China's 2007 input-output table establishment method. China Statistic Press, Beijing (2009).
6. Xin, C., Chuanmin, S., Liang, Y., Luwei, F. Market structure and price elasticity of China's iron ore imports. *Resources Science* **36,** 1915−1924 (2014) (in Chinese).
7. Feng, K. *et al*. Outsourcing CO_2 within China. *Proc. Natl Acad. Sci. USA*. **110,** 11654-11659 (2013).
8. Zhang, Y. *et al*. Temporal and spatial variations in consumption-based carbon dioxide emissions in China. *Renew. Sust. Energ. Rev.* **40,** 60−68 (2014).
9. Zhao, H. *et al*. Assessment of China's virtual air pollution transport embodied in trade by a consumption-based emission inventory. *Atmos. Chem. Phys.* **15,** 5443−5456 (2015).
10. Zhang, Y. A Study on China’s provincial CO_2 and air pollutants transportation and responsibility allocation based on provincial-global nested multi-regional input-output model. Ph.D thesis, Nanjing University, China (2015).
11. Liu, H. and Fan, X. CO_2 emissions transfer embedded in inter-regional trade in China. *Acta Ecol. Sin.* **34,** 3016−3024 (2014).
12. Xu, M., Allenby, B. & Chen, W. Energy and air emissions embodied in China−U.S. trade: eastbound assessment using adjusted bilateral trade data. *Environ. Sci. Technol.* **43,** 3378−3384 (2009).
13. Hertwich, E. G. & Peters, G. P. (2009) Carbon footprint of nations: a global, trade-linked analysis. *Environ. Sci. Technol.* **43,** 6414−6420.
14. Lenzen, M., Wood, R. & Wiedmann, T. Uncertainty analysis for multi-region input-output models - a case study of the UK's carbon footprint. *Econ. Syst. Res.* **22,** 43−63 (2010).
15. Bullard, C. W. & Sebald, A. V. Monte-carlo sensitivity analysis of input-output models. *Rev. Econ. Stst.* **70,** 708−712 (1988).
16. Yamakawa, A. & Peters, G. P. Using time-series to measure uncertainty in environmental input-output analysis. *Econ. Syst. Res.* **21,** 337−362 (2009).
17. Zhang, Q. *et al*. Asian emissions in 2006 for the NASA INTEX-B mission. *Atmos. Chem. Phys.* **9,** 5131−5151 (2009).
18. Tsinghua University. Multi-resolution emission inventory for China, http://www.meicmodel.org (last access: 1 August 2015), (2015).
19. National Bureau of Statistics of China). China energy statistical yearbook, 2008. China Statistics Press, Beijing (2008a).
20. NBSC (National Bureau of Statistics of China). China economic census yearbook, 2010. China Statistics Press, Beijing (2010).
21. Huo, H. *et al*. Examining air pollution in China using production- and consumption-based emissions accounting approaches. *Environ. Sci. Technol.* **48,** 14139−14147 (2014).
22. Guan, D., Peters, G. P., Weber, C. L. & Hubacek, K. Journey to world top emitter: An analysis of the driving forces of China's recent CO_2 emissions surge. *Geophys. Res. Lett.* **36,** L04709 (2009).
23. Zhao, H. *et al*. Environment-economy tradeoff for Beijing–Tianjin–Hebei’s exports. *Appl. Energ.* **184,** 926-935 (2016).
24. Lin, J., McElroy, M. B. & Boersma, K. Constraint of anthropogenic NOx emissions in China from

different sectors: a new methodology using multiple satellite retrievals. *Atmos. Chem. Phys.* **10,** 63−78 (2010).
25. Liu, H., Jacob, D. J., Bey, I. & Yantosca, R. M. Constraints from 210Pb and 7Be on wet deposition and transport in a global three-dimensional chemical tracer model driven by assimilated meteorological fields. *J. Geophys. Res.* **106,** D11, 12109−12128 (2001).
26. Wesely, M. L. Parameterization of surface resistances to gaseous dry deposition in regional-scale numerical models. *Atmos. Environ.* **23,** 1293−1304 (1989).
27. Browne, E. C. *et al*. Global and regional effects of the photochemistry of CH3O2NO2: evidence from ARCTAS. *Atmos. Chem. Phys.* **11,** 4209−4219 (2011).
28. Parrella, J. P. *et al*. Tropospheric bromine chemistry: implications for present and pre-industrial ozone and mercury. *Atmos. Chem. Phys.* **12,** 6723−6740 (2012).
29. Paulot, F. *et al*. Isoprene photooxidation: new insights into the production of acids and organic nitrates. *Atmos. Chem. Phys.* **9,** 1479−1501 (2009).
30. Jiang, X. *et al*. Revealing the hidden health costs embodied in Chinese exports. *Environ. Sci. Technol.* **49,** 4381−4388 (2015).
31. Lin, J. *et al*. China's international trade and air pollution in the United States. *Proc. Natl Acad. Sci. USA*. **111,** 1736−1741 (2014).
32. Keller, C. A. *et al*. HEMCO v1.0: a versatile, ESMF-compliant component for calculating emissions in atmospheric models. *Geosci. Model Dev.* **7,** 1409−1417 (2014).
33. Kharol, S. K. *et al*. Persistent sensitivity of Asian aerosol to emissions of nitrogen oxides, *Geophys. Res. Lett.* **40,** 1021−1026 (2013).
34. Megaritis, A. G., Fountoukis, C., Charalampidis, P. E., Pilinis, C. & Pandis S. N. Response of fine particulate matter concentrations to changes of emissions and temperature in Europe. *Atmos. Chem. Phys.* **13,** 3423−3443 (2013).
35. Oita, A. *et al*. Substantial nitrogen pollution embedded in international trade, *Nature Geosci.* **9,** 111−115 (2016).
36. Paulot, F. & Jacob, D. J. Hidden cost of U.S. agricultural exports: particulate matter from ammonia emissions. *Environ. Sci. Technol.* **48,** 903−908 (2014).
37. Wang, L. *et al*. Application of weather research and forecasting model with chemistry (WRF/Chem) over northern China: Sensitivity study, comparative evaluation, and policy implications. *Atmos. Environ.* **124,** 337−350 (2016).
38. Zhang, L. *et al*. Source attribution of particulate matter pollution over North China with the adjoint method. *Environ. Res. Lett.* **10,** 084011 (2015).
39. Bell, M. L., Morgenstern, R. D. & Harrington, W. Quantifying the human health benefits of air pollution policies: Review of recent studies and new directions in accountability research. *Environ. Sci. Policy* **14,** 357−368 (2011).
40. Chay, K., Dobkin, C. & Greenstone, M. The Clean Air Act of 1970 and adult mortality. *J. Risk Uncertainty* **27,** 279−300 (2003).
41. Wu, S. *et al*. Association of heart rate variability in taxi drivers with marked changes in particulate air pollution in Beijing in 2008. *Environ. Health Perspect.* **118,** 87−91 (2010).
42. van Donkelaar, A., Martin, R. V., Brauer, M. & Boys, B. L. Use of satellite observations for long-term exposure assessment of global concentrations of fine particulate matter. *Environ. Health Perspect.* **123,** 135 (2015).
43. Shindell, D. T., Lee, Y. & Faluvegi, G. Climate and health impacts of US emissions reductions consistent with 2 degree. *Nat. Clim. Change* **6,** 1−5 (2016).
44. West, J. J. *et al.* Co-benefits of mitigating global greenhouse gas emissions for future air quality and human health. *Nat. Clim. Change* **3,** 885−889 (2013).
45. Buonocore, J. J. *et al*. Health and climate benefits of different energy-efficiency and renewable energy choices. *Nat. Clim. Change* **6,** 100−105 (2015).
46. Driscoll, C. T. *et al*. US power plant carbon standards and clean air and health co-benefits. *Nat.*

Clim. Change **5,** 535−540 (2015).
47. Lelieveld, J., Evans, J. S., Fnais, M., Giannadaki, D. & Pozzer, A. The contribution of outdoor air pollution sources to premature mortality on a global scale. *Nature* **525,** 367−371 (2015).
48. Sillman, S. The relation between ozone, NOx and hydrocarbons in urban and polluted rural environments. *Atmos. Environ.* **33,** 1821−1845 (1999).
49. Xue, W. *et al*. Numerical study on the characteristics of regional transport of $PM_{2.5}$ in China. *China Environ. Sci.* **34,** 1361−1368 (2014).
50. Ying, Q., Wu, L. & Zhang, H. Local and inter-regional contributions to $PM_{2.5}$ nitrate and sulfate in China. *Atmos. Environ.* **94,** 582−592 (2014).
51. Fu, T. M., Jacob, D. J. & Heald, C. L. Aqueous-phase reactive uptake of dicarbonyls as a source of organic aerosol over eastern North America. *Atmos. Environ.* **43,** 1814−1822 (2009).
52. Heald, C. L. *et al*. Transpacific transport of Asian anthropogenic aerosols and its impact on surface air quality in the United States. *J. Geophys. Res.* **111,** D14310 (2006).
53. Park, R. J., Jacob, D. J., Chin, M. & Martin, R. V. Sources of carbonaceous aerosols over the United States and implications for natural visibility. *J. Geophys. Res.* **108,** AAC 5-1 (2003).
54. Tai, A. P. K. *et al*. Meteorological modes of variability for fine particulate matter ($PM_{2.5}$) air quality in the United States: implications for $PM_{2.5}$ sensitivity to climate change. *Atmos. Chem. Phys.* **12,** 3131−3145 (2012).
55. Auvray, M. Long-range transport to Europe: Seasonal variations and implications for the European ozone budget. *J. Geophys. Res.* **110,** D11303 (2005).
56. Guerova, G. *et al*. Impact of transatlantic transport episodes on summertime ozone in Europe. *Atmos. Chem. Phys.* **6,** 2057−2072 (2006).
57. Guerova, G. & Jones, N. A global model study of ozone enhancement during the August 2003 heat wave in Europe. *Environ. Chem.* **4,** 285−292 (2007).
58. Varotsos, K. V., Giannakopoulos, C. & Tombrou, M. Assessment of the impacts of climate change on European ozone levels. *Water Air Soil Poll.* **224,** 1596 (2013).
59. Jeong, J. I. & Park, R. J. Effects of the meteorological variability on regional air quality in East Asia. *Atmos. Environ.* **69,** 46−55 (2013).
60. Ku, B. & Park, R. J. Inverse modeling analysis of soil dust sources over East Asia. *Atmos. Environ.* **45,** 5903−5912 (2011).
61. Mu, Q. & Liao, H. Simulation of the interannual variations of aerosols in China: role of variations in meteorological parameters. *Atmos. Chem. Phys.* **14,** 9597−9612 (2014).
62. Park, R. J., Kim, M. J., Jeong, J. I., Youn, D. & Kim, S. A contribution of brown carbon aerosol to the aerosol light absorption and its radiative forcing in East Asia. *Atmos. Environ.* **44,** 1414−1421 (2010).
63. Trivitayanurak, W. *et al*. The composition and variability of atmospheric aerosol over Southeast Asia during 2008. *Atmos. Chem. Phys.* **12,** 1083−1100 (2012).
64. Croft, B., Pierce, J. R. & Martin, R. V. Interpreting aerosol lifetimes using the GEOS-Chem model and constraints from radionuclide measurements. *Atmos. Chem. Phys.* **14,** 4313−4325 (2014).
65. Ford, B. & Heald, C. L. Aerosol loading in the Southeastern United States: reconciling surface and satellite observations. *Atmos. Chem. Phys.* **13,** 9269−9283 (2013).
66. Johnson, M. S., Meskhidze, N. & Praju Kiliyanpilakkil, V. A global comparison of GEOS-Chem-predicted and remotely-sensed mineral dust aerosol optical depth and extinction profiles. *J. Adv. Model. Earth Sy.* **4,** 7001 (2012).
67. Wang, Q. *et al*. Global budget and radiative forcing of black carbon aerosol: Constraints from pole-to-pole (HIPPO) observations across the Pacific. *Journal of Geophysical Research: Atmospheres* **119,** 195−206 (2014).
68. Wang, X. *et al*. Exploiting simultaneous observational constraints on mass and absorption to estimate the global direct radiative forcing of black carbon and brown carbon. *Atmos. Chem. Phys.* **14,** 10989−11010 (2014b).

69. van Donkelaar, A. *et al*. Global estimates of fine particulate matter using a combined geophysical-statistical method with information from satellites, models, and monitors. *Environ. Sci. Technol.* **50,** 3762−3772 (2016).
70. Boys, B. L. *et al*. Fifteen-year global time series of satellite-derived fine particulate matter. *Environ. Sci. Technol.* **48,** 11109−11118 (2014).
71. Lim, S.S. *et al*. A comparative risk assessment of burden of disease and injury attributable to 67 risk factors and risk factor clusters in 21 regions, 1990–2010: a systematic analysis for the Global Burden of Disease Study 2010. *Lancet* **380,** 2224−2260 (2012).
72. Brauer, M. *et al*. Exposure assessment for estimation of the global burden of disease attributable to outdoor air pollution. *Environ. Sci. Technol.* **46,** 652−660 (2012).
73. Dockery, D. W. *et al*. An association between air pollution and mortality in six US cities. *New Engl. J. Med.* **329,** 1753−1759 (1993).
74. Pope III, C. A. *et al*. Lung cancer, cardiopulmonary mortality, and long-term exposure to fine particulate air pollution. *J. Am. Med. Assoc.* **287,** 1132−1141 (2002).
75. Brook, R. D. *et al*. Particulate matter air pollution and cardiovascular disease: An update to the scientific statement from the American Heart Association. *Circulation* **121,** 2331−2378 (2010).
76. Chen, H., Goldberg, M. S. & Villeneuve, P. J. A systematic review of the relation between long-term exposure to ambient air pollution and chronic diseases. *Rev. Environ. Health* **23,** 243−298 (2008).
77. Bell, M. L., McDermott, A., Zeger, S. L., Samet, J. M. & Dominici, F. Ozone and short-term mortality in 95 US urban communities, 1987-2000. *J. Am. Med. Assoc.* **292,** 2372−2378 (2004).
78. Jerrett, M. *et al*. Long-term ozone exposure and mortality. *New Engl. J. Med.* **360,** 1085−1095 (2009).
79. Burnett, R. T. *et al*. An integrated risk function for estimating the global burden of disease attributable to ambient fine particulate matter exposure. *Environ. Health Perspect.* **122,** 397−403 (2014).
80. Kinney, P. L. *et al*. On the use of expert judgment to characterize uncertainties in the health benefits of regulatory controls of particulate matter. *Environ. Sci. Policy* **13,** 434−443 (2010).
81. Roman, H. A. *et al*. Expert judgment assessment of the mortality impact of changes in ambient fine particulate matter in the US. *Environ. Sci. Technol.* **42,** 2268−2274 (2008).
82. Ostro, B. *et al*. Associations of mortality with long-term exposures to fine and ultrafine particles, species and sources: results from the California Teachers Study Cohort. *Environ. Health Perspect.* **123,** 549−556 (2015).
83. Arnold, C. Disease burdens associated with $PM_{2.5}$ exposure: how a new model provided global estimates. *Environ. Health Perspect.* **122,** A111 (2014).
84. Lei, Y., Zhang, Q., He, K. & Streets D. G. Primary anthropogenic aerosol emission trends for China, 1990-2005. *Atmos. Chem. Phys.* **11,** 931−954 (2011).
